# Proteomics discovery of MTDH and SND1 interaction vulnerabilities in ovarian cancer

**DOI:** 10.1038/s41598-025-26913-1

**Published:** 2025-11-24

**Authors:** Parisa Esmaeili, Ahmad Nasimian, Lucas Werner, Sergio Mosquim Junior, Magnus E. Jakobsson, Anna Sandström Gerdtsson, Julhash U. Kazi, Fredrik Levander

**Affiliations:** 1 Department of Immunotechnology, Lund University, Lund, 223 81 Sweden; 2 Division of Translational Cancer Research, Department of Laboratory Medicine, Lund University, Lund, Sweden; 3 Department of Biomedical Science, Faculty of Health and Society, Malmö University, Malmö, 20506 Sweden; 4 Lund Stem Cell Center, Department of Laboratory Medicine, Lund University, Lund, Sweden; 5 Science for Life Laboratory, National Bioinformatics Infrastructure Sweden, Lund University, Lund, Sweden

**Keywords:** Cancer, Cell biology, Computational biology and bioinformatics, Drug discovery

## Abstract

High-grade serous ovarian cancer (HGSOC) is the most prevalent and aggressive subtype of ovarian cancer. The combination of late-stage diagnosis and the tendency to exhibit resistance to existing treatments highlights a critical gap in effective therapeutic options. There is thus a need for novel strategies for targeting HGSOC, particularly in its advanced stages. To address this gap, we developed a comprehensive atlas profiling both the proteome and phosphoproteome levels across a panel of nine ovarian cancer cell lines from different subtypes. Subsequent differential expression analysis between the KURAMOCHI and the other cell lines, followed by phosphosite analyses, proposed the MTDH protein as a potential target. Further functional analyses of MTDH and the interacting protein SND1 with RNA silencing, as well as targeting their interaction, revealed that disrupting this interaction leads to the dysregulation of several pathways associated with cancer progression and invasion. In particular, interference with the MTDH-SND1 complex was associated with enrichment of ferroptosis-related pathways. Moreover, combining C26A6 treatment with ferroptosis inducers produced enhanced inhibitory effects in ovarian cancer cells, suggesting a possible strategy for targeting cancer cell vulnerabilities in HGSOC, which warrants further investigation beyond in vitro models.

**Supplementary Information:**

The online version contains supplementary material available at 10.1038/s41598-025-26913-1.

## Introduction

Ovarian cancer stands as the most lethal form of gynecologic malignancy affecting women worldwide^1^. This disease exhibits remarkable heterogeneity, characterized by various types and subtypes. Epithelial ovarian cancer (EOC), which represents approximately 90% of all ovarian cancer cases, is categorized into five distinct histological subtypes. Among these, high-grade serous ovarian carcinoma (HGSOC) is predominant, accounting for 70% of EOC cases. It is typically diagnosed at an advanced stage, leading to the majority of ovarian cancer-related deaths^2,3^. HGSOC is notable for its high fatality rate, highlighting the critical need for improved treatment strategies.

Ovarian cancer is commonly diagnosed after it has already metastasized to the abdominal cavity, a stage at which the disease becomes particularly challenging to treat. Despite initial responsiveness to standard treatments, which include surgery and platinum-based chemotherapy, there is an alarming 80% relapse rate due to chemoresistance^2,4^. The absence of effective therapies for metastatic stages and chemoresistance represents the primary obstacles to decreasing the mortality rate associated with metastatic ovarian cancer^5,6^. Addressing this gap calls for the adoption of high-throughput technologies, such as omics, to explore new biomarkers and decode the complex mechanisms of drug resistance.

Among omics technologies for biomarker discovery, proteomics offers distinct advantages over genomics and transcriptomics in cancer research due to its direct assessment of proteins and their posttranslational modifications (PTMs). PTMs are the ultimate effectors of cellular function and are not detectable by genomics-based approaches^7,8^. Mass spectrometry-based proteomics stands out for its high analytical depth and quantitative ability to identify protein biomarkers^9^. Additionally, phosphoproteomics, achieved through the enrichment of phosphopeptides and measurements using the same technology, plays a pivotal role in monitoring the regulation of essential cancer hallmarks such as cell migration, invasion, and metastasis. Abnormalities in phosphorylation of key proteins within these processes can pinpoint vulnerabilities in cancer cells that, when targeted, could offer promising new treatment avenues^10^.

The primary goal of this study was to identify dysregulated mechanisms within ovarian cancer cells to discover new therapeutic entry points that can lead to more efficient and personalized treatment options. As fundament, we first created a comprehensive atlas of protein and phosphoprotein profiles from nine ovarian cancer cell lines from different subtypes, using liquid-chromatography-mass spectrometry (LC-MS) in data-independent acquisition (DIA) mode. Exploration of these datasets facilitates not only discovery of regulation differences in proteins and phosphopeptides between cell lines representing different subtypes, but also showcases the diversity across different cell lines, even from the same subtype.

We then performed comparative proteomics and phosphoproteomics analyses between the KURAMOCHI cell line, noted for its genetic similarity to tumor tissue^11,12^, and other ovarian cancer cell lines. This approach was used to identify dysregulated pathways and prioritize candidate proteins for further investigation, ultimately leading us to focus on MTDH and SND1, two proteins known for their roles in metastasis and drug resistance in breast cancer^13^, which we investigated in the context of ovarian cancer. Our analysis indicates that the interaction of these two proteins is likely to be relevant to ovarian cancer progression and merits further investigation as a possible therapeutic target.

## Results

### Mapping the proteomic and phosphoproteomic landscape to identify therapeutic targets in ovarian cancer

**Fig. 1:**
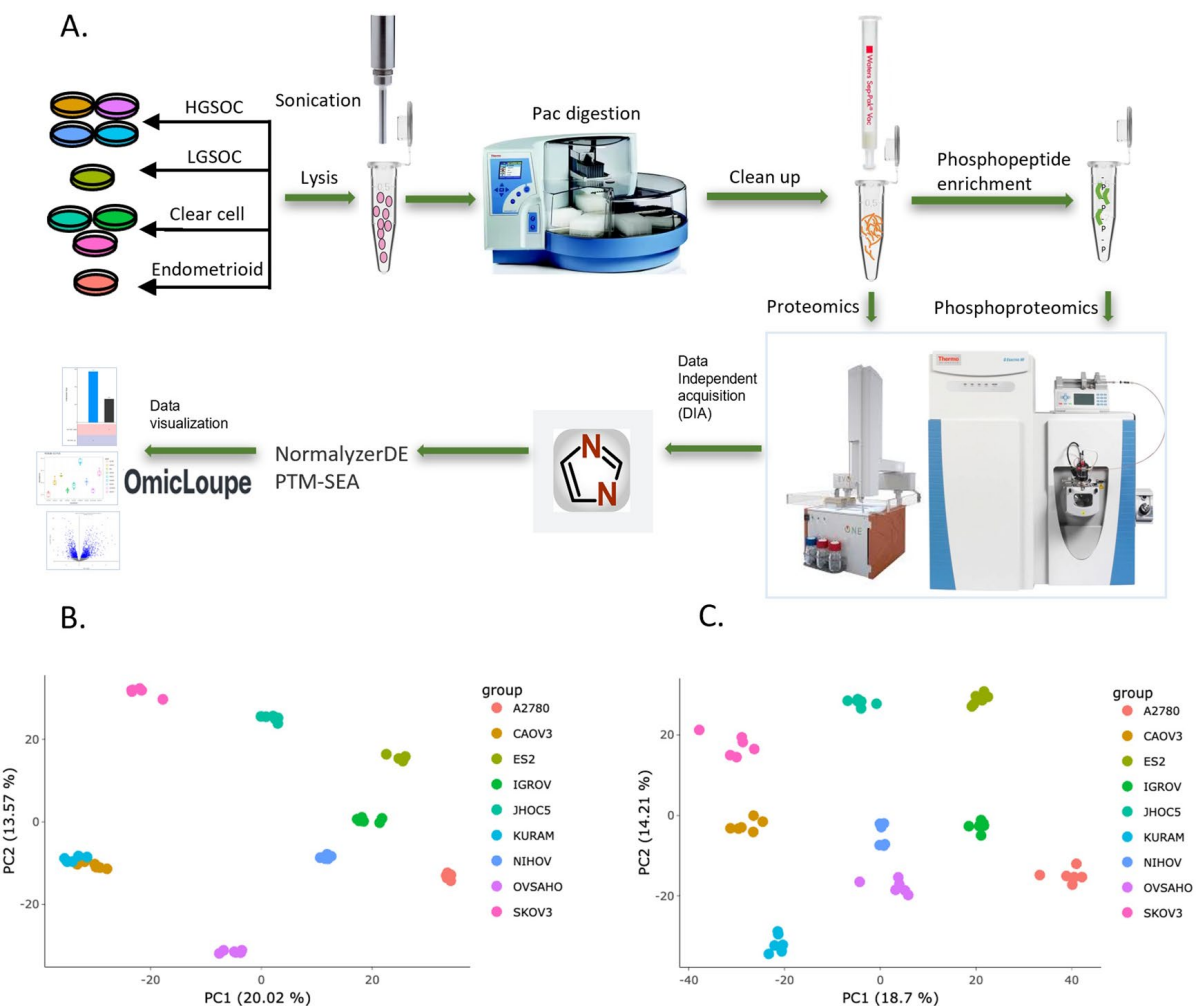
Overview of proteomics and phosphoproteomics analyses**. A** Schematic workflow diagram depicting the experimental procedures for proteomics and phosphoproteomics. **B** PCA of the proteomics data for nine ovarian cancer cell lines with six replicates. **C** PCA of the phosphoproteomics data. Principal components (PC) 1 and 2 are plotted. Color coding for cell lines is used consistently across all panels to ensure clear differentiation.

To characterize the proteomic and phosphoproteomic landscapes of ovarian cancers, we performed a comprehensive analysis using LC-MS DIA across a selection of ovarian cancer cell lines, each representing distinct subtypes and genetic alterations (Fig. 1A). The panel of cell lines used in this study includes well-established ovarian cancer models with diverse genetic backgrounds^11^. It encompassed four major ovarian cancer subtypes: HGSOC—KURAMOCHI, NIHOVCAR3, OVSAHO, and CAOV3 cell lines; clear cell carcinoma—SKOV3, IGROV1, and JHOC5 cell lines; endometrioid carcinoma—A2780 cell line; and low-grade serous ovarian cancer—ES2 cell line^14^.

We quantified 7153 protein groups and 28,475 phosphopeptides in the proteomics and phosphoproteomics analyses, respectively, and subsequently applied principal component analysis (PCA) on the six replicates of all cell lines to reduce dimensionality. All replicates within each cell line formed distinct clusters, regardless of whether proteomic (Fig. 1B) or phosphoproteomic (Fig. 1C) data were analyzed. This clustering highlights the high reproducibility of the experimental procedures but also the complexity of ovarian cancer biology with large differences between the cell lines. Moreover, distinct separation was observed at both proteome and phosphoproteome levels between the cell lines. Furthermore, groups of cell lines representing the same subtypes could be distinguished in the PCA plots, aligning with their identified subtypes (Supplementary Fig. 1). We have deposited the proteomic data in a publicly available repository to allow for further investigation of cell line-specific proteome and phosphoproteome attributes^15^.

### Enriched proteomic features of aggressiveness in KURAMOCHI

**Fig. 2:**
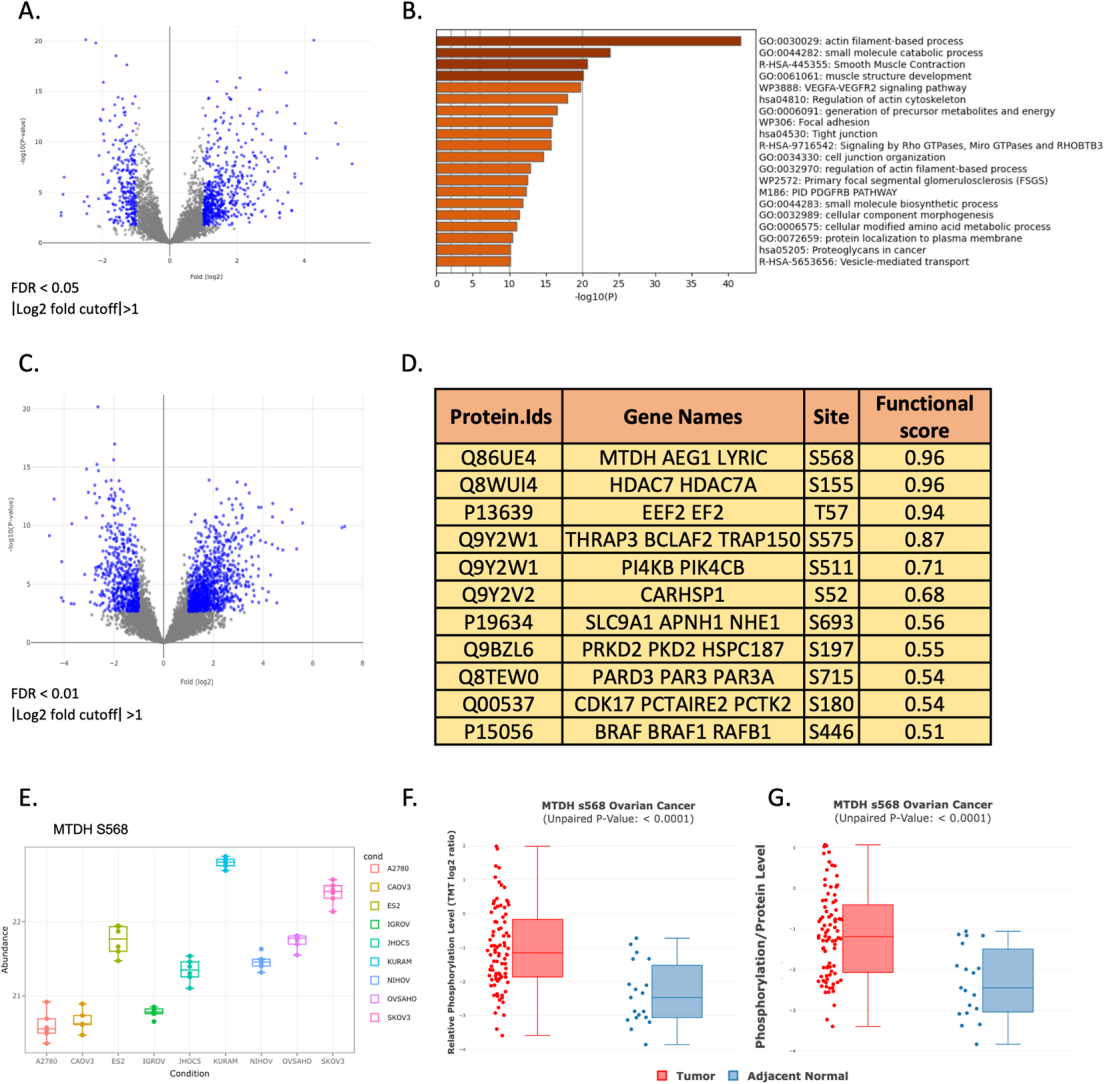
Proteomic and phosphoproteomic analysis of the KURAMOCHI cell line compared with eight other ovarian cancer cell lines. **A** Volcano plot displaying the comparative protein profile analysis of KURAMOCHI against the other cell lines, highlighting proteins with FDR < 0.05 and |log2 fold change| > 1. The positive fold changes correspond to higher levels in KURAMOCHI. **B** Bar chart showing the enriched gene ontologies and pathways in KURAMOCHI, analyzed using Metascore. **C** Volcano plot illustrating the phosphoproteomic comparison, focusing on phosphoproteins with FDR < 0.01 and |log2 fold change| > 1. **D **Functional analysis of phosphosites using phosphosite functional scores. **E** Box plot demonstrating the expression levels of the MTDH S568 phosphopeptide across all analyzed cell lines. Y-axis abundance values represent log2-transformed, normalized intensity. **F,G** Phosphorylation of MTDH S568 in tumor and adjacent healthy tissues. Data were obtained from the cProSite proteomic dataset, with statistical significance indicated above the plots. **F** Relative phosphorylation levels of MTDH S568. **G** Phosphorylation-to-protein ratio of MTDH S568.

Given the apparent variation in proteomes between the cell lines, with many proteins differing between individual and groups of cell lines, we set out to explore those differences further, knowing that the large heterogeneity of ovarian cancers sets a need for precision treatment. The KURAMOCHI cell line has been shown to have strong genomic resemblance to patient-derived HGSOC tumors, based on profiling of gene expression, mutations, and copy-number alterations^11,12^. KURAMOCHI obtained the highest suitability score as cell line model for HGSOC in the study of Domcke et al. ^11^ as it is closely resembling patient-derived HGSOC tumors in terms of copy-number alterations, mutation frequency and mutation status in key genes. It has mutations in *TP53* and *BRCA2*, amplifications in *MYC* and *KRAS*, and exhibits functional homologous recombination deficiency, making it highly suitable as an HGSOC model^11,16^. We therefore initially compared the proteome and phosphoproteome of this cell line to those of the other eight ovarian cancer cell lines. The protein expression profile displayed 842 proteins with significant differences (FDR < 0.05 and log2 fold change > 1) when comparing it to the other cell lines (Fig. 2A, Supplementary Data S1). Among those 576 proteins were detected at significantly higher abundance in KURAMOCHI, while 266 proteins were found at lower levels. We subjected the proteins detected at higher levels to enrichment analyses in Metascape^17^ (Fig. 2B). Several gene ontologies and pathways were found to be enriched, with actin filament-based processes, small molecule catabolic processes, and smooth muscle contraction being the most significant. These enriched functions and processes are consistent with features often associated with enhanced motility and aggressiveness in cancer cells, suggesting that the proteomic profile of KURAMOCHI may reflect a more mobile phenotype with metastatic potential.

### High level of phosphorylated MTDH in KURAMOCHI

Protein phosphorylation is a critical regulatory mechanism, and analysis of the phosphorylation status highlights how proteins interact with and activate various signaling pathways, thereby offering a more nuanced understanding of cellular function and the molecular underpinnings of cancer. We, therefore, analyzed the differentially abundant phosphopeptides between the KURAMOCHI cell line and the other cell lines (FDR < 0.01 and log2 fold change > 1), revealing 1096 phosphopeptides with significantly higher abundance and 604 with significantly lower levels in KURAMOCHI cells (Fig. 2C, Supplementary Data S2). To prioritize phosphosites with potential biological relevance, we applied a functional score analysis as described by Ochoa et al.^18^. This scoring system integrates evolutionary conservation, structural context, and functional annotation to predict the likelihood that a given phosphosite is functionally important. We focused this analysis on the differentially expressed phosphosites, particularly those with increased abundance in KURAMOCHI cells. Through this method, we could identify the phosphosites with the highest functional scores (Fig. 2D), including Metadherin (MTDH at S568), Histone Deacetylase 7 (HDAC7 at S155), and Eukaryotic Elongation Factor 2 (EEF2 at T57). MTDH is involved in various cellular processes, including oncogenesis, metastasis, and chemotherapy response^19^; HDAC7 plays a role in transcriptional regulation, development, and cell cycle progression through histone deacetylation^20^; EEF2 is crucial for protein synthesis, facilitating the translocation of ribosomes along mRNA^21^. Abundance of the MTDH S568 peptide varied across the cell lines, with KURAMOCHI showing the highest levels (Fig. 2E). To explore the levels of this phosphorylation in tumors and adjacent tissue, we explored clinical phosphoproteomics data from the Clinical Cancer Proteomics Consortium (CPTAC) using cProSite^22^. MTDH S568 phosphorylation showed a significant increase in tumor samples compared to adjacent normal tissues ( p-value < 0.0001, Fig. 2F), with a large spread across the analyzed tumors. Furthermore, the phosphorylation-to-protein ratio at this site was significantly higher in tumors, suggesting a tumor-specific enhancement of this post-translational modification (p-value < 0.0001, Fig. 2G).

### MTDH overexpression and its functional link to SND1 in ovarian cancer

**Fig. 3:**
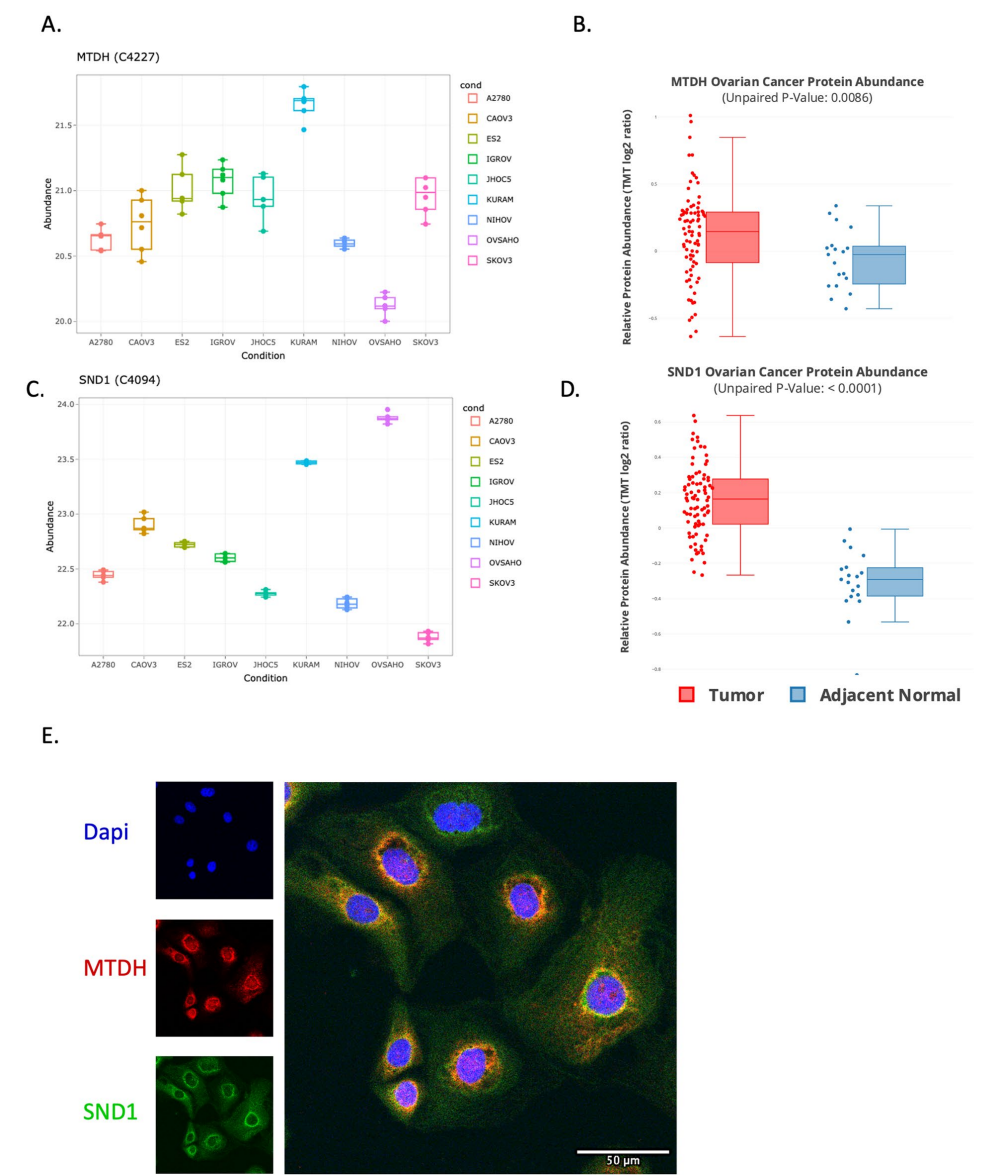
Expression levels of MTDH and SND1. **A**,**C** Expression levels of MTDH (**A**) and SND1 (**C**) in ovariancancer cell lines. Abundance represent log2-transformed normalized protein intensities. Statistical analysisshowed that MTDH expression in Kuramochi compared with other cell lines was highly significant (adjusted pvalue= 6E-7), and SND1 expression was also significantly different (adjusted *P*-value = 0.003). **B** cProSiteproteomic analysis shows significantly higher MTDH expression in tumor samples compared to adjacentnormal tissues (*P*-value < 0.05). **D** cProSite proteomic analysis shows significantly higher SND1 expression intumor samples compared to adjacent normal tissues (*P*-value < 0.05). **E** Immunocytochemistry showing MTDH(red) and SND1 (green), with nuclei stained blue; scale bar = 50 μm.

Following our phosphoproteomic analysis, MTDH emerged as an interesting candidate based on its high functional score and elevated phosphorylation at S568 in KURAMOCHI cells. Notably, the MTDH protein itself was also found at high levels in KURAMOCHI, showing a similar pattern to the S568 phosphosite across the panel of ovarian cancer cell lines, though with slightly less variation between the highest and lowest levels (Fig. 3A). Furthermore, proteomic data from cProSite, showed significantly higher MTDH expression in ovarian tumors compared to adjacent normal tissues (p-value < 0.008, Fig. 3B).

These findings prompted us to explore MTDH in greater depth as a potential oncogenic driver in HGSOC. Previous studies have implicated MTDH in tumor progression, metastasis, and poor clinical outcomes across various cancer types. Among its known interaction partners, SND1 has been described as a key effector of MTDH-mediated oncogenic signaling, particularly in the context of metastasis and chemoresistance^13,23–25^. The protein levels of SND1 across the cell lines in this study were highest in OVSAHO and KURAMOCHI (Fig. 3C). Similarly to MTDH, analysis of cProSite data indicated significantly higher levels of SND1 expression in ovarian tumor tissues relative to adjacent normal tissues (p-value < 0.0001, Fig. 3D). Together, these observations highlighted the MTDH–SND1 axis as a potentially important mechanism in ovarian cancer, emerging from our integrated proteomic and phosphoproteomic analysis. This led us to experimentally investigate the localization and functional roles of both proteins in relevant cell models.

To characterize the cellular localization of MTDH and SND1 we performed immunocytochemical analyses of both KURAMOCHI and OVSAHO cells. MTDH was predominantly localized to the perinuclear region of the nucleus and was also detected within the cytoplasm (Fig. 3E, Supplementary Fig. 2). Similarly, protein SND1 was observed primarily at the nuclear periphery, with substantial distribution throughout the cytoplasm, indicating a dual compartmental role for these proteins. The cytoplasmic distribution of MTDH in endometrial and ovarian cancer has also been observed by Meng et al. ^26^. In the cytoplasm, MTDH associates with RNA and RNA-binding proteins, including SND1 ^26^. This interaction is crucial for MTDH’s role in promoting cell survival and drug resistance, positioning it as a potential therapeutic target^25,26^. This observation is in line with our experimental findings in ovarian cancer cell lines, further supporting the oncogenic role of SND1.

### Dose-dependent response to drug targeting of the MTDH-SND1 interaction

**Fig. 4:**
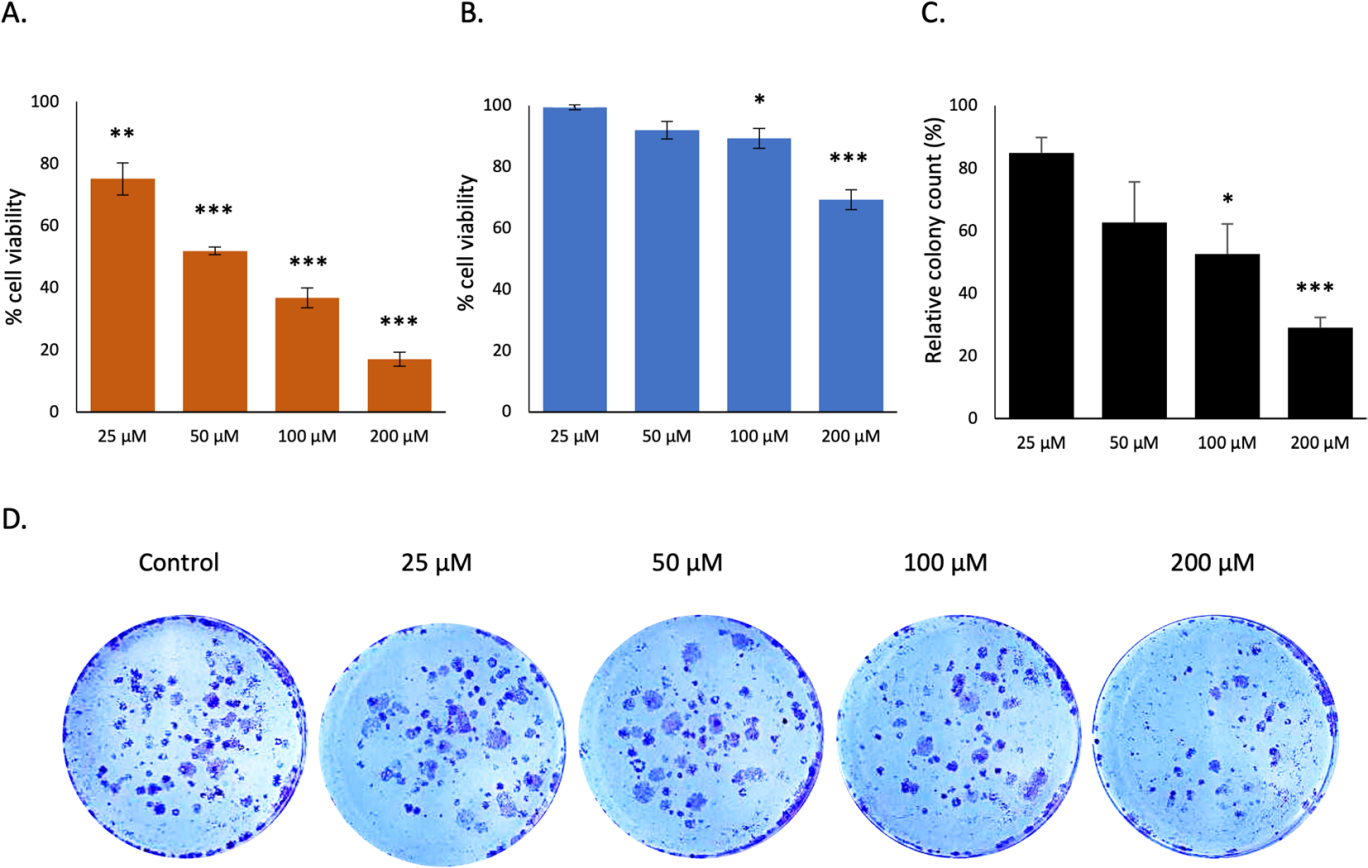
Cellular assays for C26A6. **A** PrestoBlue and **B** ATPlite cell viability assays measuring the effects of C26A6 treatment over 72 h. Cell viability values were normalized to the untreated control and expressed as percentage (%). Each assay was performed in triplicate; error bars represent SEM (*n* = 3), **C**,** D** Colony formation assay on cells treated with C26A6 for 3 days and monitored for 10 days. Colonies were fixed, stained, counted, and normalized to the untreated control. **C** Bar graph shows relative colony formation (%) compared to control; error bars represent SEM (*n* = 3). **D** Wells after 10 days. Statistical significance was determined using an unpaired two-tailed Welch’s t-test of luminescence or colony counts in comparison with controls. Significance levels are indicated as: *P* < 0.05 (*), *P* < 0.01 (**), *P* < 0.001 (***).

The compound C26A6 has been reported as a potent inhibitor of metastasis, and enhanced chemotherapy sensitivity by disrupting the interaction between MTDH and SND1 proteins in breast cancer^13^. We treated KURAMOCHI with C26A6 and assessed cell viability using the ATP and PrestoBlue assays. The results demonstrated a dose-dependent response to the compound, cell viability decreased with increasing drug concentration (Fig. 4A, B). There were differences between the two assays, although both are used to measure cell viability through different mechanisms. The ATP Lite assay quantifies cellular activity by measuring ATP levels, indicative of cell viability and energy production. Conversely, the PrestoBlue assay, a resazurin-based compound, measures metabolic activity, offering insights into cellular health but not necessarily aligning with viable cell counts^27^. We also evaluated the colony-forming ability of the cells after treatment. The results demonstrated a dose-dependent response to C26A6, affecting the colony formation of the cells (Fig. 4C, D).

### Proteome and phosphoproteome effects of targeting MTDH–SND1

**Fig. 5:**
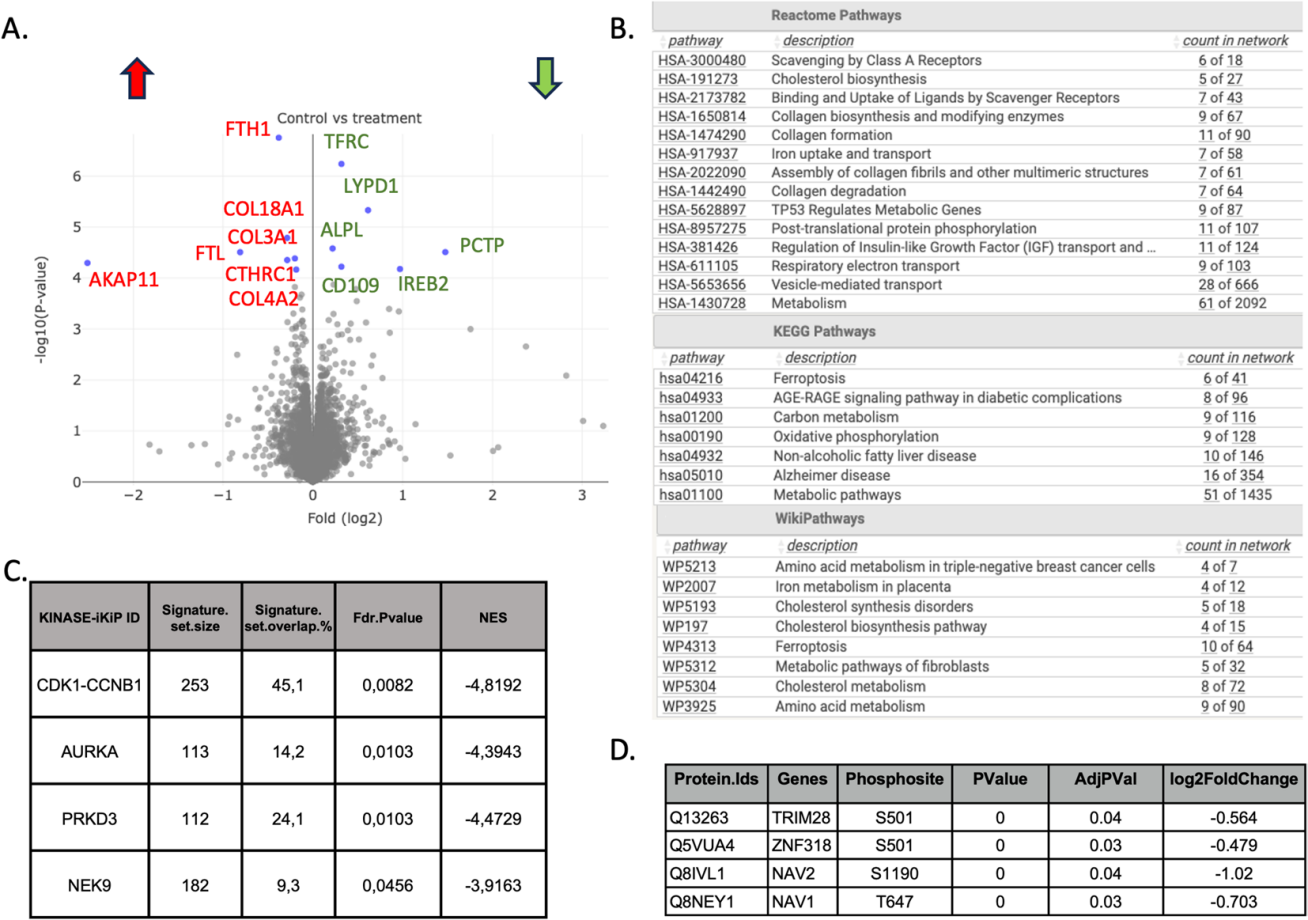
Proteomic and phosphoproteomic analysis following targeting of the MTDH-SND1interaction with C26A6. **A** Identification of significantly differentially expressed genes (FDR < 0.05), the red arrow indicates upregulated proteins in treated samples, while the green arrow denotes downregulated proteins. **B** Functional enrichment analysis of differentially expressed genes (p-value < 0.05) in STRING using multiple databases. **C** PTM Signature Enrichment Analysis (PTM-SEA) following treatment, focusing on kinase substrate targets (Kinase iKiP sets). NES is the Normalized Enrichment Score. **D** significantly downregulated phosphopeptides (FDR < 0.05).

While the disruption of the SND1–MTDH interaction has been previously characterized in breast cancer, its downstream effects in ovarian cancer remain largely unexplored. Here, we aimed to investigate proteome- and phosphoproteome-level changes following inhibition of this interaction in ovarian cancer cells to uncover potential mechanisms of action. Based on viability assays, we selected 50 µM C26A6 as a sub-cytotoxic concentration for treatment. At this dose, PrestoBlue results indicated approximately 50% viability, while ATP-Lite measurements showed ~ 90% viability, suggesting that the compound induces early metabolic effects before overt cell death. This concentration was therefore chosen to capture molecular alterations associated with MTDH–SND1 inhibition, minimizing confounding effects from widespread cytotoxicity. Following this treatment, a total of 6,026 proteins were quantified, of which 13 were significantly differentially abundant at FDR < 0.05 (Fig. 5A). Significantly downregulated genes upon treatment were LYPD1, ALPL, IREB2, CD109, and TFRC which each has shown effect in metastasis and tumorigenesis^28–32^. The treatment of cancer cells with C26A6 led to significant changes in the protein abundance across various key biological pathways. To detect pathway level differences after C26A6 treatment, also among proteins that showed less pronounced differences, differentially abundant proteins (371 proteins, p-value < 0.05, Supplementary Data S3) were subjected to functional enrichment analysis in STRING and demonstrated distinct pathway enrichments across multiple databases. Specifically, ferroptosis pathways were found to be significantly enriched in both the KEGG and WikiPathways databases. Collagen formation pathways were notably highlighted within the Reactome database, while amino acid metabolism showed enrichment in WikiPathways (Fig. 5B). The enrichment of ferroptosis pathways suggests that the drug may induce cell death through iron-dependent oxidative stress, a mechanism that is increasingly recognized for its potential in cancer therapy^33^. Additionally, the alteration of collagen formation pathways could imply impacts on the extracellular matrix and tumor microenvironment, potentially affecting tumor stiffness and metastatic potential^34^. The observed changes in amino acid metabolism and general metabolic pathways indicate a broad alteration in cellular metabolism, which may reflect the cells’ adaptive responses to the drug or a direct effect of the drug itself on metabolic processes. These insights collectively suggest that blocking MTDH-SND1 using C26A6 exerts multifaceted effects on cancer cells, disrupting primary metabolic functions and specific pathways critical to cancer cell survival and proliferation.

In the phosphoproteomic analysis of the cells, 14,793 phosphopeptides were identified, of which 845 were found to be differentially abundant (p-value < 0.05) when treated with C26A6 (Supplementary Data S4). Phosphosite signature enrichment analysis (PTM-SEA)^35^ was performed to identify differentially phosphorylated kinase substrates^36^. The analysis revealed a downregulation in phosphorylation levels of kinase substrates, indicating reduced kinase activity of CDK1-CCNB1, AURKA, PRKD3, and NEK9 with FDR < 0.05 (Fig. 5C). Four phosphopeptides showed significantly lower abundance after treatment (FDR < 0.05, Fig. 5D). The findings demonstrate the drug’s precise targeting of cell proliferation and invasion in cancer cells. The downregulated kinases are intricately linked to crucial cellular processes such as survival, progression, and proliferation.

### Silencing of MTDH and SND1 shows distinct and common effects

To unveil the intrinsic molecular mechanisms and cellular processes involving MTDH and SND1, and excluding potential off-target effects of C26A6, we transfected KURAMOCHI cells with siRNA targeting each protein separately and examined the resulting proteome and phosphoproteome profiles (Supplementary Fig. 3). The proteins with increased or decreased abundance (FDR < 0.01, Supplementary Data S5, S6) were analyzed for molecular function and pathway overlaps^37,38^. The silencing of MTDH significantly lowered proteins found in gene sets involved in cancer-related pathways such as VEGFA-VEGFR2 and EGF-EGFR signaling (Table 1), which are crucial for angiogenesis and cell proliferation. Additionally, observed reductions in protein levels in pathways essential for actin cytoskeleton regulation and integrin-mediated cell adhesion suggest a role for MTDH in enhancing cancer cell motility and invasion. Decreased levels of proteins involved in molecular functions such as cadherin-, cytoskeletal protein-cell adhesion molecule- and purine nucleotide-binding were observed in both MTDH and SND1 silencing. The silencing of SND1 led to a marked downregulation of proteins in both the VEGFA-VEGFR2 and EGF-EGFR signaling pathways, similar to the effects observed with MTDH. Additionally, lowered levels of proteins involved in the pleural mesothelioma pathway were found, highlighting this protein’s potential role in mesothelial cell tumorigenesis (Table 1). Moreover, the molecular function and pathway overlap analysis of upregulated proteins showed several shared entities in invasion and cell proliferation upon silencing of the two proteins separately (Supplementary Table 1). The observed mix of upregulation and downregulation among cancer-related functions underscores the nuanced compensatory mechanisms initiated by cells in response to gene silencing and may reflect a concerted effort to restore internal equilibrium. Collectively, these findings emphasize the shared role of MTDH and SND1 in promoting cancer progression and highlight their potential as therapeutic targets.

We further conducted PTM-SEA using phosphoproteomics data from the siRNA transfections (Supplementary Table 2). Notably, the silencing of both genes led to significant decreases in phosphosite signatures of CK2A2 and CK2A1 inhibition. Both are catalytic subunits of the protein kinase CK2 (casein kinase 2), a serine/threonine-protein kinase. CK2 is implicated in various cellular functions and tumorigenesis, with elevated activity linked to malignant tissue transformation and aggressive tumor behavior^39^.

**Table. 1:**
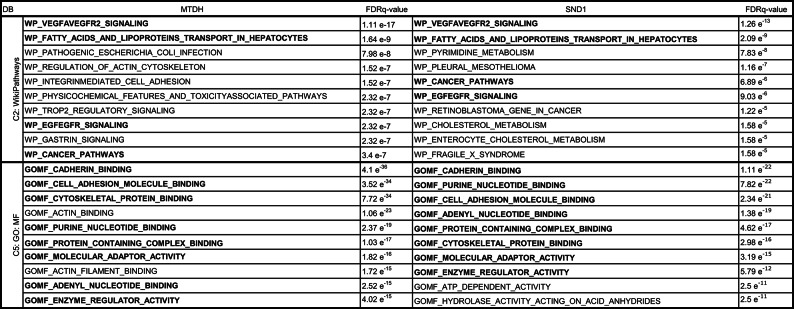
Pathway over-representation analysis performed using the MSigDB overlap tool following MTDH and SND1 silencing, . using the top 500 down-regulated proteins after filtering at FDR < 0.01. DB = Database. Bold text indicates pathways that are present in both MTDH and SND1 silenced samples.

### C26A6 enhances susceptibility to ferroptosis inducers and inhibits ovarian cancer cell proliferation

**Fig. 6:**
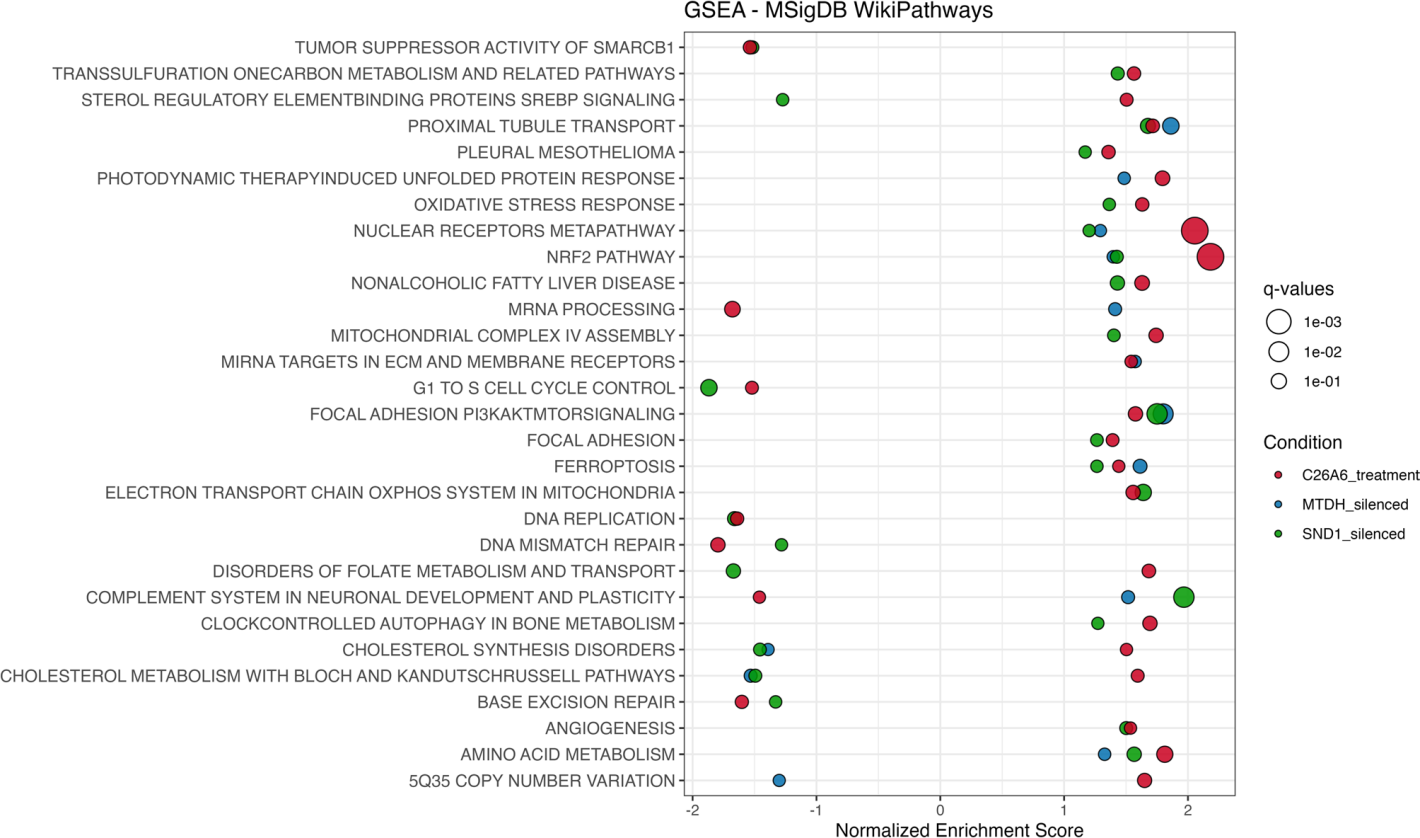
GSEA using WikiPathways for pathways commonly enriched in both C26A6-treated samples and those after MTDH or SND1 silencing. Shared pathways (FDR < 0.5) are displayed to highlight consistent responses. Bubble size represents significance (q-values represents FDR adjusted p-values).

To identify overlapping biological pathways affected by C26A6 treatment and silencing of MTDH and/or SND1, we performed pathway enrichment analysis based on differentially expressed proteins. We then filtered the results to include only those pathways that were shared between C26A6 treatment and at least one of the silencing conditions (MTDH, SND1, or both). These shared pathways were visualized using a bubble plot (Fig. 6). Several key pathways were consistently enriched, including ferroptosis, proximal tubule transport, nuclear receptors metapathway, NRF2 pathway, focal adhesion–PI3K–AKT–mTOR signaling, complement system, cholesterol synthesis and metabolism, and amino acid metabolism. These overlaps suggest that targeting MTDH–SND1 affects broad cellular processes, with ferroptosis being one of the shared pathways. The enrichment of overlapping pathways across all three conditions suggests that these are not incidental findings. Rather, the overlap suggests that these pathways represent true downstream consequences of targeting the MTDH–SND1 axis, reinforcing the biological relevance of this interaction in ovarian cancer cell regulation.

**Fig. 7:**
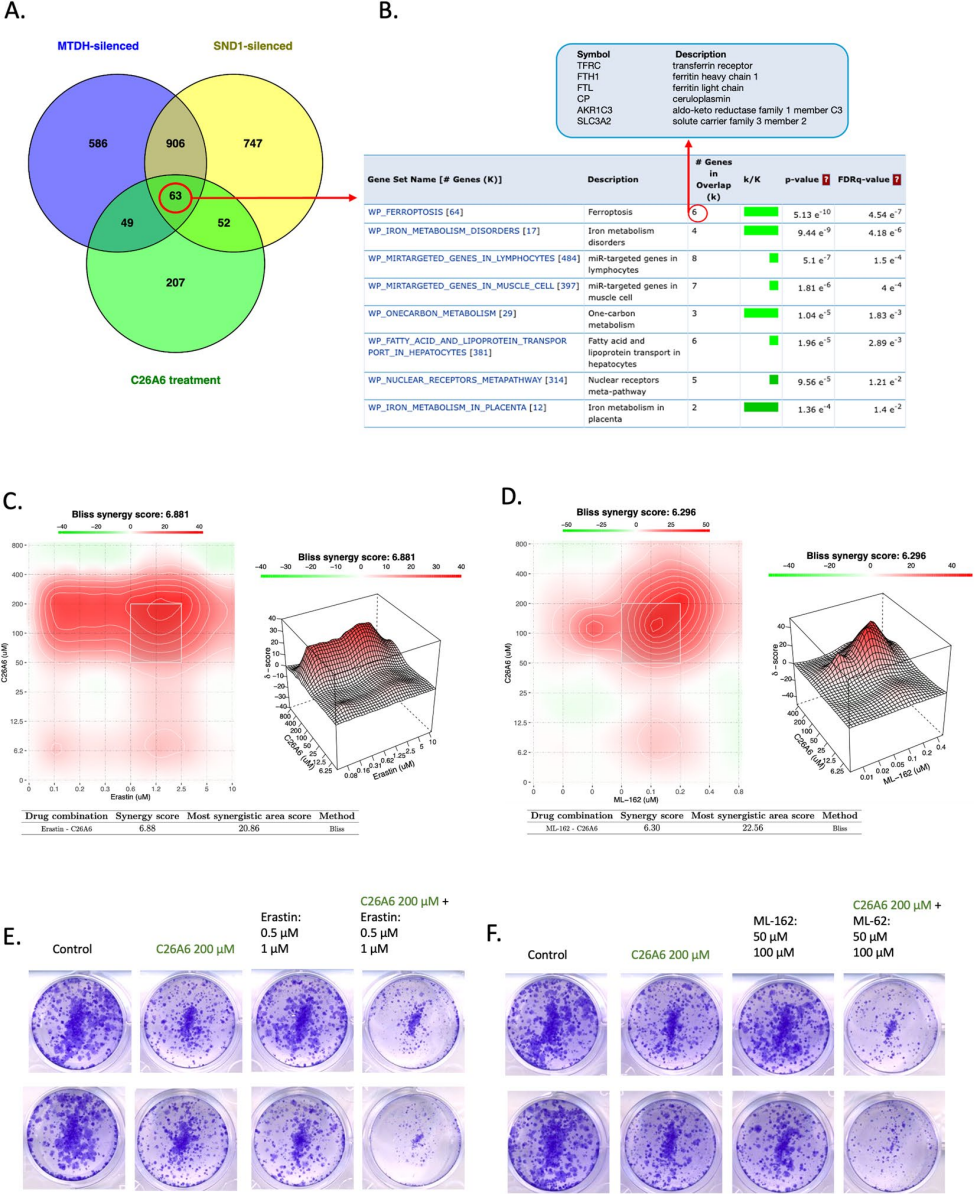
Overlap of differentially abundant proteins in C26A6-treatmented and SND1- and MTDH-silenced groups highlights ferroptosis. **A** Venn diagram (Venny) showing shared differentially expressed proteins between C26A6-treatment (p-value < 0.05) and MTDH- or SND1-silencing (FDR < 0.05). **B** Enriched pathways identified through over-representation analysis based on the shared protein sets. Ferroptosis is among the key pathways consistently enriched across all conditions, with six overlapping proteins involved. **C**,** D** Synergy analysis of C26A6 combined with **C** Erastin or **D** ML-164 treatment in the KURAMOCHI cell line. Cell viability was calculated relative to DMSO-treated control cells and measured using the CellTiter-Glo assay over 5 days of treatment. **E**,** F** Colony formation assay of C26A6 (200 µM) combined with **E** Erastin (0.5 µM and 1 µM) or **F** ML-162 (50 nM and 100 nM) treatments after 3 days and monitored for up to 10 days.

Interestingly, pathway enrichment analysis in WikiPathways of 63 common proteins that were significantly differentially abundant upon C26A6-treatment and MTDH- and SND1-silencing (Fig. 7A, Supplementary Data S7) demonstrated enrichment in ferroptosis pathway and iron metabolism (Fig. 7B), consistent with previous GSEA results. Among the overlapping proteins were six key ferroptosis-related proteins: TFRC, FTH1, FTL, CP, AKR1C3, and SLC3A2, highlighting a potential regulatory link between MTDH–SND1 inhibition and ferroptotic vulnerability. Given that C26A6 exhibited dysregulation in the ferroptosis process, we investigated its interaction with two ferroptosis inducers, Erastin and ML-162, to evaluate their combined effects with C26A6 on HGSOC cell lines. Combination effects between the drugs were assessed using SynergyFinder^40^, indicating a synergistic effect with a Bliss score of 6.9 in the combination with Erastin and a Bliss score of 6.3 in the combination with ML-162 in the KURAMOCHI cell line (Fig. 7C, D). Notably, SynergyFinder also reported “most synergistic area” scores of ~ 21 for both combinations, indicating strong synergy at specific dose ranges. Next, we applied the same combined treatments to evaluate their impact on the colony-forming ability and observed a synergistic decrease in colony formation (Fig. 7E, F). To explore the broader applicability of our findings, we also tested CAOV3, which lacks a BRCA mutation but harbors a TP53 alteration with moderate HGSOC resemblance, and IGROV1, which carries a BRCA1 mutation but exhibits a hypermutated, non-HGSOC molecular profile^11^ (Supplementary Figs. 4, 5). The results in both showed Bliss scores > 0, indicating synergy^41^, although at varying levels. Based on these observations, our data suggest that C26A6 reduces cell proliferation and may increase cellular susceptibility to ferroptosis inducers, resulting in enhanced inhibitory effects on ovarian cancer cells.

## Discussion

Ovarian cancer is a heterogeneous disease comprising several distinct subtypes, each with unique molecular characteristics. Despite originating in the same location, ovarian cancer subtypes exhibit diverse molecular profiles, contributing to variations in disease behavior and treatment responses^42^. In this study, we initially created an atlas of different ovarian cancer subtypes. Subsequently, we employed a representative cell line model^11^ to investigate specific proteins as potential therapeutic targets.

The proteomics results suggest that KURAMOCHI cells exhibit a more aggressive phenotype compared to the other cells. At the pathway level, this was indicated through the increased abundance of proteins in the actin cytoskeleton and proteins involved in smooth muscle cell migration, which is a mechanism that plays a pivotal role in the spread of tumors through metastasis^43,44^. Furthermore, the presence of pathways such as VEGFA signaling and regulation of focal adhesion in the enrichment analysis, points to differences in cell growth, movement, and intercellular interactions^45,46^.

We found that MTDH was highly abundant in KURAMOCHI compared to other cell lines, and complementary analysis using the cProSite resource indicated that MTDH expression is significantly elevated in ovarian tumor tissue relative to adjacent normal tissue. Previous research has revealed that MTDH possesses the capacity to interact with various proteins, thereby facilitating the assembly of multiprotein complexes^47^. The significant role of MTDH in the advancement and formation of mammary cancers has been highlighted by Wan et al., 2014. This was particularly noted in its influence on the proliferation and function of tumor-initiating cells (TICs)^23^. Other research has found that high levels of MTDH are associated with worse outcomes and higher chances of recurrence in various cancers, including breast, bladder, colon, and hepatocellular carcinoma, underscoring its role in cancer progression and patient prognosis^13,24,48,49^. Furthermore, various studies have identified SND1 as a crucial binding partner of MTDH, highlighting its significant role in cancer metastasis and chemoresistance. The interaction between MTDH and SND1 is essential for the function of MTDH in tumor-initiating cells, suggesting their pivotal role in cancer progression and potential as therapeutic targets^13,23–25^. In the present study, although we did not perform co-immunoprecipitation in ovarian cancer cells, we provide complementary evidence through immunofluorescence staining and confocal microscopy, demonstrating colocalization of MTDH and SND1 (Fig. 3E). Consistent with this, cProSite data also showed that SND1 abundance is higher in ovarian tumor tissue compared with adjacent normal tissue (Fig. 3D), reinforcing its potential role in ovarian cancer biology. Both MTDH and SND1 were also highly expressed in KURAMOCHI cells. Together, these findings, alongside published evidence, underscore the relevance of the MTDH–SND1 interaction in cancer biology and provided the rationale for focusing on this interaction in our study. Using the MTDH-SND1 inhibitor (C26A6), we showed the dose-dependent response in cell-based assays. The greater reduction in PrestoBlue compared to ATP suggests that the compound rapidly impairs cellular metabolic activity, while short-term ATP levels are partly maintained, potentially through metabolic compensation. However, the dose-dependent decrease in colony formation indicates that this compensation is insufficient to sustain long-term proliferative capacity.

We employed a low dose of C26A6 to observe its initial effects on the proteome of cancer cells. While the MTDH–SND1 interaction and the effects of C26A6 have been studied in breast cancer^13^, this is, to our knowledge, the first evaluation of this pharmacologic interaction inhibitor in ovarian cancer. Inhibition of the MTDH-SND1 interaction presents significant changes in iron metabolism, specifically ferroptosis. In proteomics data, proteins associated with ferroptosis and iron metabolism, such as the Transferrin Receptor (TFRC), Ferritin Heavy Chain 1 (FTH1) and Ferritin Light Chain (FTL) were notably altered in the context of MTDH and SND1 silencing (Supplementary Fig. 6). High TFRC levels link to worse outcomes in EOC, and reducing TFRC can significantly decrease EOC cell growth and spread^28^. Our results show that disrupting the MTDH-SND1 interaction greatly lowers TFRC expression (Fig. 5A). Moreover, we detected decreased levels of kinases involved in cell cycle regulation and cellular processes. Additionally, we observed downregulation of pathways related to cell growth and cell cycle both at the proteome and phosphoproteome levels when silencing MTDH and SND1 (Table 1, Supplementary Tables 1–2).

In GSEA of proteomic data from samples treated with C26A6, Nuclear factor erythroid 2–related factor 2 (NRF2) emerged as the most enriched pathway (Fig. 6, Supplementary Fig. 7). NRF2 is crucial in regulating ferroptosis due to its multifaceted roles in iron, lipid, and amino acid metabolism^50^. Consistent with this, NRF2 pathway enrichment was also observed following MTDH silencing and SND1 silencing, and our shared pathway analysis (Fig. 6) demonstrated convergence on amino acid metabolism and related processes. These findings further support a mechanistic link between disruption of the MTDH–SND1 axis and increased ferroptosis susceptibility. A recent study revealed that elevated levels of MTDH inhibit ferroptosis, and this increase in MTDH expression paradoxically made the cancer cells more sensitive to ferroptosis-inducing treatments^51^. Comparable to that study, we also found that SLC3A2 was significantly more abundant following both C26A6 treatment and MTDH RNA silencing. The GPX4 protein median abundance also showed a similar trend, although not statistically significant. (Supplementary Fig. 8). This dual role highlights MTDH as a candidate for further evaluation as a therapeutic biomarker. Whereas that study focused on MTDH expression levels as determinants of susceptibility; here, we demonstrate that disruption of the MTDH–SND1 interaction itself—achieved by siRNA-mediated knockdown of MTDH or SND1, or by C26A6 treatment—enhances ferroptosis pathway enrichment. Notably, C26A6 did not significantly alter total MTDH protein levels (Supplementary Fig. 9), indicating that the observed effects are driven by interaction disruption rather than changes in protein abundance. We cannot, however, exclude the possibility that some of the proteomic changes observed after C26A6 treatment may reflect off-target or general cytotoxic effects. Nevertheless, the similarity between treatment and genetic silencing increases confidence that the major findings are linked to disruption of the MTDH–SND1 axis.

The combination of C26A6 with either Erastin (system X_c_^-^ inhibitor)^33^ and ML-162 (GPX4 inhibitor)^51^ showed a synergistic effect in different ovarian cancer cell lines (Supplementary Fig. 4,5). Specifically, high levels of MTDH might identify patients who would benefit most from ferroptosis-based therapies, as these cells, despite their resistance mechanism, could be more effectively targeted and killed by treatments designed to induce ferroptosis^51,52^. Furthermore, recent research highlights targeting ferroptosis as a promising strategy to overcome chemotherapy resistance in ovarian cancer^53^. Our findings suggest that C26A6, even at a low dose, regulates ferroptosis, the NRF2 pathway, iron metabolism, and amino acid metabolism. This multifaceted regulation suggests that combining C26A6 with ferroptosis inducers could be explored as a strategy to enhance therapeutic efficacy against HGSOC.

In conclusion, our study highlights the heterogeneity of ovarian cancer and identifies MTDH as a protein of interest in its progression. Disruption of the MTDH–SND1 interaction using the compound C26A6 was associated with alterations in iron metabolism and ferroptosis-related proteins. Importantly, in vitro combination experiments with ferroptosis inducers showed enhanced inhibitory effects across multiple ovarian cancer cell lines, suggesting that interference with this interaction may increase susceptibility to ferroptosis. While these findings are preliminary and limited to cell models, they provide a basis for further preclinical studies to explore the therapeutic relevance of targeting the MTDH–SND1 axis in ovarian cancer.

## Materials and methods

### Cell lines and cell culture

Human ovarian cancer cell lines CAOV3, NIHOVCAR3, ES2, SKOV3, A2780, IGROV1, OVSAHO, KURAMOCHI and JHOC5, were cultured under conditions designed to mimic their physiological environment. CAOV3 cells were maintained in DMEM supplemented with GlutaMAX™, 10% fetal bovine serum (FBS), and 1% penicillin/streptomycin (P/S). NIHOVCAR3 required RPMI medium enriched with GlutaMAX™, a higher concentration of FBS (20%), 1% P/S, and 0.01 mg/mL sterile-filtered bovine insulin to support its insulin-responsive growth traits. ES2 and SKOV3 lines were both cultured in McCoy’s 5 A medium with GlutaMAX™, 10% FBS, and 1% P/S. A2780 and IGROV1 cells were grown in DMEM with GlutaMAX™, 10% FBS, 1% P/S, supplemented with 1x non-essential amino acids (NEAA) and 1x MEM vitamin solution to support their comprehensive metabolic needs. OVSAHO, and KURAMOCHI cell lines were cultured in RPMI medium with GlutaMAX™, supplemented with 10% FBS and 1% P/S. JHOC5 cells were grown in DMEM/F12 medium with GlutaMAX™, 10% FBS, 1% P/S, and 1x NEAA to cater to their specific growth requirements. All cell lines were incubated at 37 °C in a humidified 5% CO2 atmosphere. Regular mycoplasma testing was conducted to ensure the absence of contamination. Cell cultures were passaged upon reaching approximately 80–90% confluency, ensuring continuous growth and viability for experimental use. Mouse ovarian cancer cell lines PPNM and BPPNM cells were maintained in DMEM supplemented with GlutaMAX^TM^, with 1% Insulin-Transferrin-Selenium (Gibco ITS-G, 41400045), 100 µl EGF (10ug/ml), 4% FBS, and 1% P/S.

The cell lines CAOV3, NIHOVCAR3, ES2, OVSAHO, KURAMOCHI, and JHOC5 were obtained from the laboratory of Dr. Ingrid Hedenfalk (Lund University, Sweden). SKOV3, A2780, and IGROV1 were purchased from Sigma-Aldrich. All cell lines were tested for mycoplasma contamination before use and maintained under standard culture conditions.

### Cell viability assays

To assess cell viability using the PrestoBlue assay (Fisher Scientific GTF, A13261), Cells were seeded in 96-well plates (Corning) at densities optimized for each cell line: KURAMOCHI, 1,500 cells/well; Caov-3, 2,000 cells/well; and IGROV-1, 1,500 cells/well. Plates were incubated overnight to allow cell attachment before treatment.After treatment with drug, the cells were incubated for 3–5 days. At the end of the incubation, 10 ul PrestoBlue reagent was added directly to the cells without a medium change, and the plates were incubated for 30 min, typically 10 min to 2 h depending on the cell type, to allow viable cells to reduce the PrestoBlue reagent, resulting in a fluorescent signal. The fluorescence intensity, proportional to the number of living cells, was measured using a fluorescence microplate reader at an excitation and emission wavelength, of around 560/590 nm. This method offers a quick and sensitive measure of cell viability and cytotoxicity. Other assays for measuring cell viability were the ATPlite luminescence assay (PerkinElmer, 6016943) and CellTiter-Glo (Promega Biotech, G9242). Briefly, 30 µL of cell lysis solution was added to each well and incubated for 5 min to lyse the cells and release intracellular ATP. Subsequently, 30 µL of the substrate solution containing luciferase and luciferin was added to each well, and the plate was gently shaken for 5 min to ensure thorough mixing. The luminescence, which is proportional to the amount of ATP present, was measured using a microplate reader. All experiments were performed in triplicate, and the data were expressed as mean ± standard deviation. Drugs used were Erastin (Fisher Scientific GTFHY-15763), ML-162 (Nordic Biosite − 20455), and C26A6 (Biotechne − 7692).

### Colony formation assay

Cells were seeded in 12-well plates at the following densities: KURAMOCHI, 500 cells/well (single-agent) or 2,000 cells/well (combination), IGROV-1, 2,000 cells/well, and Caov-3, 5,000 cells/well. After overnight attachment, cells were treated for 3 days, then cultured in drug-free medium for 10 days. Colonies were fixed and stained with 2–3 mL of 6.0% glutaraldehyde/0.5% crystal violet for 30 min, rinsed with water, and air-dried before imaging.

### Synergy calculations

To evaluate the synergistic effects of drug combinations, we tested eight doses of each drug individually and in combination, with each condition performed in triplicate. We utilized DECREASE^54^, (a machine learning-based method) for predicting drug combination dose-response landscapes with minimal experimental data. Experimental validation was conducted using the CellTiter-Glo (Promega Biotech AB- G9242). Synergy scores were calculated using the Bliss independence model via SynergyFinder^40^, a web application for analyzing drug combination dose-response matrix data. Synergistic interactions were quantified, with Bliss synergy scores greater than zero indicating synergy.

### Transfection

Cells were seeded at a density of 100,000 cells per well in a 6-well plate and allowed to reach 60–80% confluency. Before transfection, the media was refreshed. Transfection reagent Lipofectamine (13778150, Thermo fisher Lipofectamine™ RNAiMAX) was diluted in 150 µl of medium (9 µl per sample) to prepare a master mix. Each sample, including siRNA targeting specific genes and negative controls, was prepared by adding 30 pmol of siRNA (Sigma- EHU024601 MISSION esiRNA targeting human MTDH, EHU054581 MISSION esiRNA targeting human SND1and SIC001 MISSION siRNA Universal NegativeControl1), to 150 µl of fresh media. A 1:1 ratio of the master mix was added to each corresponding tube containing siRNA. The mixture was incubated for 5 min at room temperature to allow complex formation. Subsequently, 250 µl of the siRNA-Lipofectamine complex was added to each well. The media was replaced between 6- and 24 h post-transfection, and cells were harvested 72 h after transfection for further analysis.

### Immunocytochemistry

Cells were seeded overnight in cell culture slides (Falcon Chambered Cell Culture Slides), fixed with 4% paraformaldehyde, permeabilized with 0.1% Triton X-100, and blocked in 5% BSA. They were incubated with primary antibodies against MTDH (Abcam, ab227981) and SND1(Proteintech, CL594-60265) proteins overnight at 4 °C, followed by incubation with fluorophore-conjugated secondary antibody (Abcam-Goat Anti-Rabbit, ab150083) for 1 h at room temperature. Nuclei were stained with DAPI (Thermo Fisher, D1306). Images were captured using Confocal fluorescence microscope (Zeiss 710).

### Western blot

Protein extracts from transfected and control cells were quantified using the BCA assay (ThermoFisher). Equal amounts of protein were separated on SDS-PAGE (ThermoFisher) and then transferred to PVDF membranes (Sigma). The membranes were blocked with 5% BSA in TBST for 1 h at room temperature, followed by incubation with primary antibodies MTDH, and SND1 overnight at 4 °C. After washing, membranes were incubated with HRP-conjugated secondary antibodies for 1 h at room temperature. Protein bands were visualized using an enhanced chemiluminescence (Merck). β-Actin (AH diagnostic, sc-47778) was used as the loading control. Band intensities were analyzed using ImageJ.

### Protein extraction and digestion

Cells at 70–80% confluency were lysed using boiling lysing buffer (5% SDS (Thermo Fisher Scientific, Vilnius, Lithuania), 0.1 M TRIS pH 8.5 (Thermo Scientific, Rockford, IL), 10 mM 2-CAA (Sigma-Aldrich, St. Louis, MO) and 5 mM TCEP (Thermo Scientific, Rockford, IL) dissolved in LC/MS grade dH2O (Fischer chemical, Ottawa) and transferred into low protein binding tubes. The lysates were then sonicated with a microtip for 2 min (1 s on/1 s off pulses, 50% amplitude) on ice. Protein concentrations were estimated using BCA assay. PAC digestion (protein aggregation capture)^55^ of the lysates was performed with a mixture of LysC (1:500) and trypsin (1:250). This was automated on a KingFisher™ Flex Robot (Thermo Fisher Scientific), utilizing MagResyn^®^ hydroxyl beads (Resyn Biosciences) in 96-well plates for bead-based digestion. Digestion was quenched by adding TFA to a final concentration of 1% post-completion of the process. Following digestion, peptides were desalted using 50 mg SepPak tC18 Vac Cartridge columns (Waters, Milford, MA).

### Phosphopeptide enrichment

Phosphorylated peptides were enriched using the KingFisher Flex Robot along with MagResyn^®^ Ti-IMAC HP beads (Resyn Biosciences). This automated process was executed in 96-well plates following a specific 8-step protocol designed for phosphoproteomic analysis. Briefly, the beads were equilibrated in a binding solvent composed of 80% MeCN, 5% TFA, and 0.1 M glycolic acid (Sigma-Aldrich, St. Louis, MO). Peptides, initially dissolved in 0.1% TFA at 4 µg/µL and then diluted in the same binding solvent, were bound to these equilibrated beads. Post-binding, the beads were washed sequentially with more of the binding solvent, followed by 80% MeCN + 1% TFA, and then 10% MeCN + 0.2% TFA. Phosphopeptides were eluted with 1% ammonia (pH 10) and immediately acidified using TFA to a final concentration of 1%.

### LC-MS

Peptides and phosphopeptides were analyzed using an Evosep One (Evosep) Q Exactive HF-X Orbitrap (Thermo Scientific) LC-MS system. Specifically, 600 ng of peptides or all enriched phosphopeptides, redissolved in 20 µL of 0.1% formic acid, were loaded onto conditioned Evotips (Evosep) for desalting, and initial offset before column injection. The chromatographic separation in the initial cell line landscape analysis was performed on a C18 Evo column (EV1109, Evosep) using a 21-minute gradient, allowing for 60 samples per day (SPD). For cell treatment and transfection experiments, an in-house packed PicoFrit column was used with the 58 min whisper 20 SPD method for full proteomes samples, and 31 min 40 SPD whisper for phosphopeptide samples. Mass spectrometry analysis was performed using data-independent acquisition (DIA). For 21 min runs the DIA method used an MS1 scan range from 350 to 1400 m/z, 120,000 resolution, maximum 25 ms injection time with 3E6 target, MS/MS used default charge 2, loop count 49, 15,000 resolution, 13.7 m/z isolation windows, NCE 27, 22 ms maximum injection time with 3E6 target, with DIA window centers from 471.5 to 1129.1 m/z. Profile mode was used for both for MS1 and MS2. For 58 min runs MS1 scans were from 395 to 1005 m/z, 60,000 resolution, 55 ms maximum injection time with 3E6 target, with centroid acquisition. For MS/MS default charge 3, resolution 15,000, Auto maximum injection time with 1E6 target, NCE27, loop count 75, 8 m/z windows, with a total of 151 staggered windows with centers between 400.4 and 1000.7 m/z. For 31 min runs MS1 scans were from 350 to 1400 m/z at 120,000 resolution, 25 ms maximum injection time with 3E6 target. For MS/MS default charge 2, 15,000 resolution, auto maximum injection time with 3E6 target, loop count 50, 100 staggered 13.7 m/z windows between 471.5 and 1149.65 m/z. All spectra for the 31 min and 58 min methods were collected in centroid mode.

#### Data processing

MS raw data files were converted to mzML using msconvert of Proteowizard^56^ with vendor peak picking for profile mode files and demultiplexing for files with staggered DIA windows. DIA-NN 1.8.1^57^ in library-free mode was used for processing of the data into quantitative peptide and protein tables. The data were searched with the reviewed part of the human proteome UniProtKB database as of May 2022 with max 1 missed cleavage and fixed cysteine carbamidomethylation. Full proteome data were searched with no variable modification. Phosphoenriched data were searched with max one variable phospho (STY) for the cell line landscape data and max two phospho (STY) for the treatment data. The data were filtered to 1% false discovery rate (FDR) at the peptide and protein level within DIA-NN.

### Data analysis

Normalization of the protein and phosphopeptides abundance data and differential expression analysis using LIMMA ebayes statistics were conducted using NormalyzerDE v1.20^58^. Benjamini Hochberg correction of p-values was used for filtering of group-wise differential expression comparisons at different FDRs. OmicLoupe^59^ was used for the generation of Principal Component Analysis (PCA), volcano and box plots. Enrichment analyses were performed using STRING^60^, Metascape^17^ and Gene set enrichment analysis (GSEA)^37,38^ with ClusterProfiler^61^ to identify enriched pathways and biological processes, providing insights into the functional significance of the data.

Functional scores for phosphosites were calculated using the funscoR R package^18^(version 0.1.0), Briefly, phosphosites were annotated using annotate_sites(), and separate functional models were trained for serine/threonine (ST) and tyrosine (Y) residues using a gold-standard dataset derived from PhosphoSitePlus (psp). Features were preprocessed, and scores were predicted using predict_funscore(). The resulting functional scores were log-transformed and used to prioritize biologically relevant phosphorylation sites. This approach is conceptually based on the framework described by Ochoa et al^18^, but scores were computed specifically for our dataset using custom-trained models. PTM signature enrichment analysis (PTM-SEA) was performed using PTMSigDB human flanking sequence signatures v2.0^35^.

## Supplementary Information

Below is the link to the electronic supplementary material.

Supplementary Material 1

Supplementary Material 2

## Data Availability

The raw data mass spectrometry data and the DIA-NN search results have been deposited to the ProteomeXchange Consortium via the PRIDE partner repository with the dataset identifier PXD059892, available at https://www.ebi.ac.uk/pride/archive/projects/PXD059892.
