# Supplementary material for: Proteomics discovery of MTDH and SND1 interaction vulnerabilities in ovarian cancer
Source: Sci Rep. 2025 Nov 24;15:41818. doi: 10.1038/s41598-025-26913-1 (PMC12647655; doi:10.1038/s41598-025-26913-1)
Supplement: Supplementary file 2 — Supplementary Material 2 [file 41598_2025_26913_MOESM2_ESM.pdf]

Supplemental information

# Proteomics discovery of MTDH and SND1 interaction vulnerabilities in ovarian cancer

Parisa Esmaeili, Ahmad Nasimian, Lucas Werner, Sergio Mosquim Junior, Magnus E. Jakobsson, Anna Sandström Gerdtsson, Julhash U. Kazi and Fredrik Levander

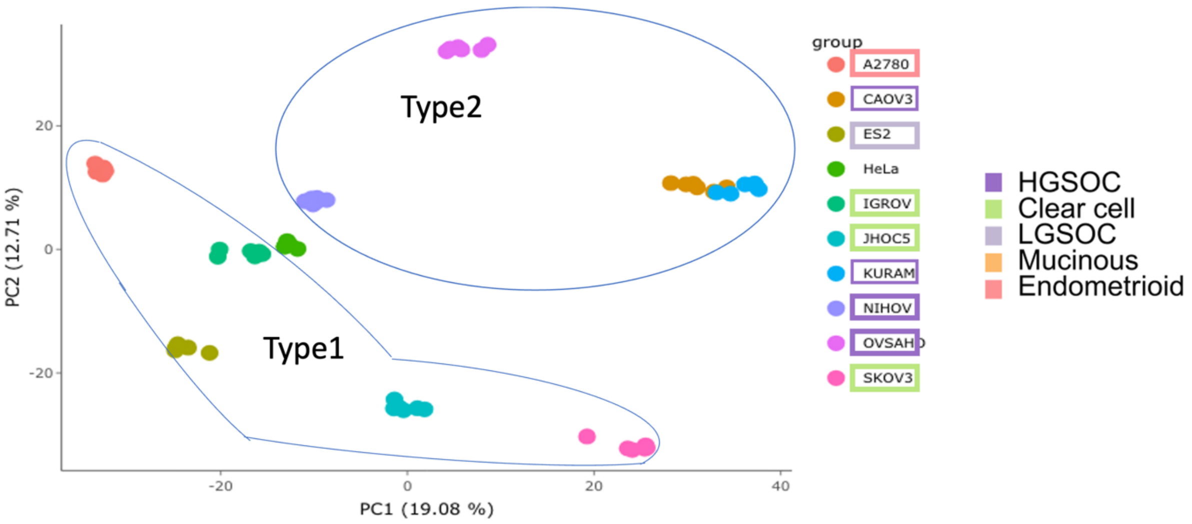


**Supplementary Figure. 1** PCA of nine ovarian cancer cell lines within each subtype. Type 1 includes less aggressive subtypes and Type 2 includes highly aggressive subtypes.

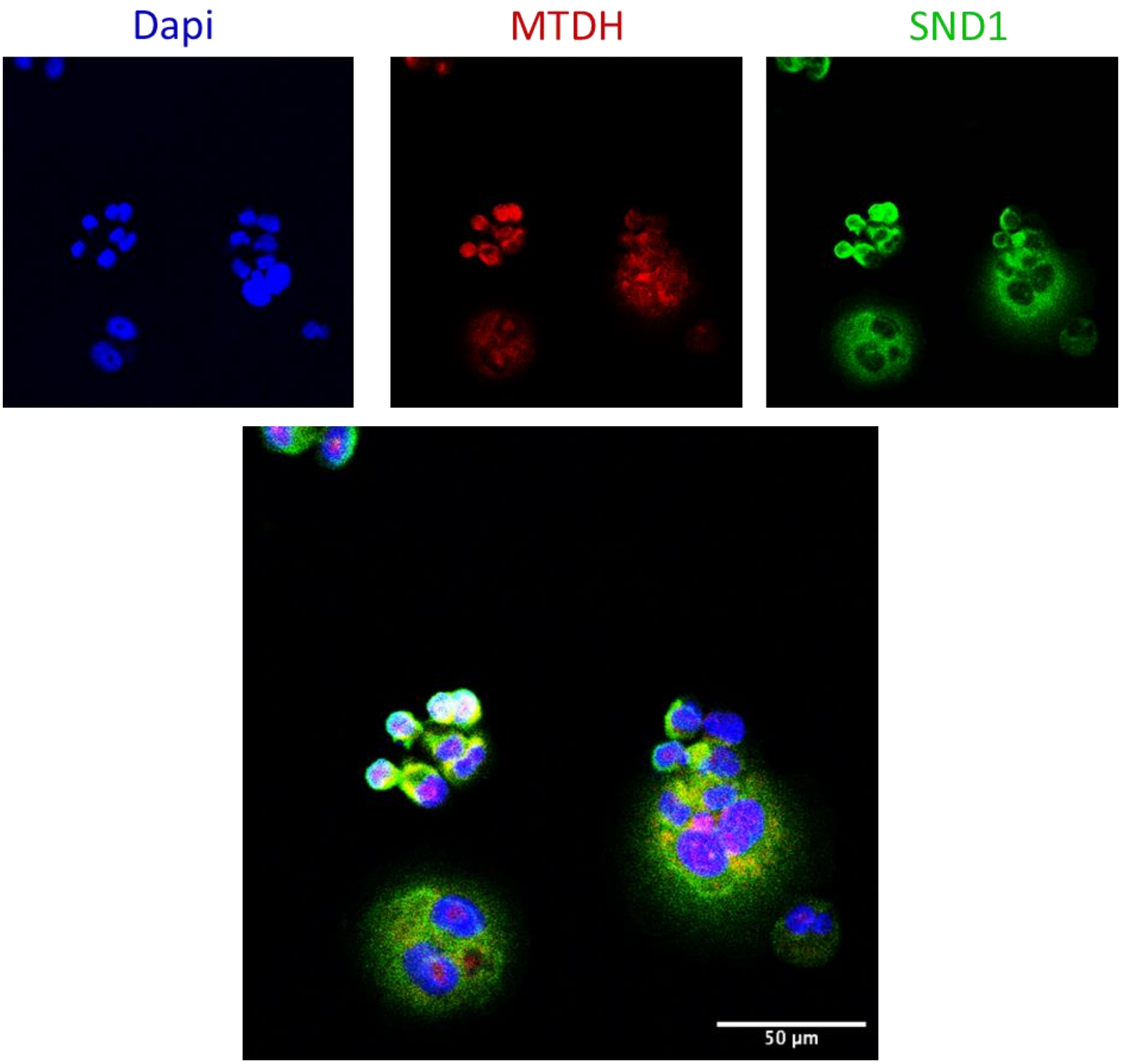


**Supplementary Figure 2.** Immunocytochemistry in OVSAHO cell line, showing MTDH (red) and SND1 (green), with nuclei stained blue; scale bar = 50 μm.

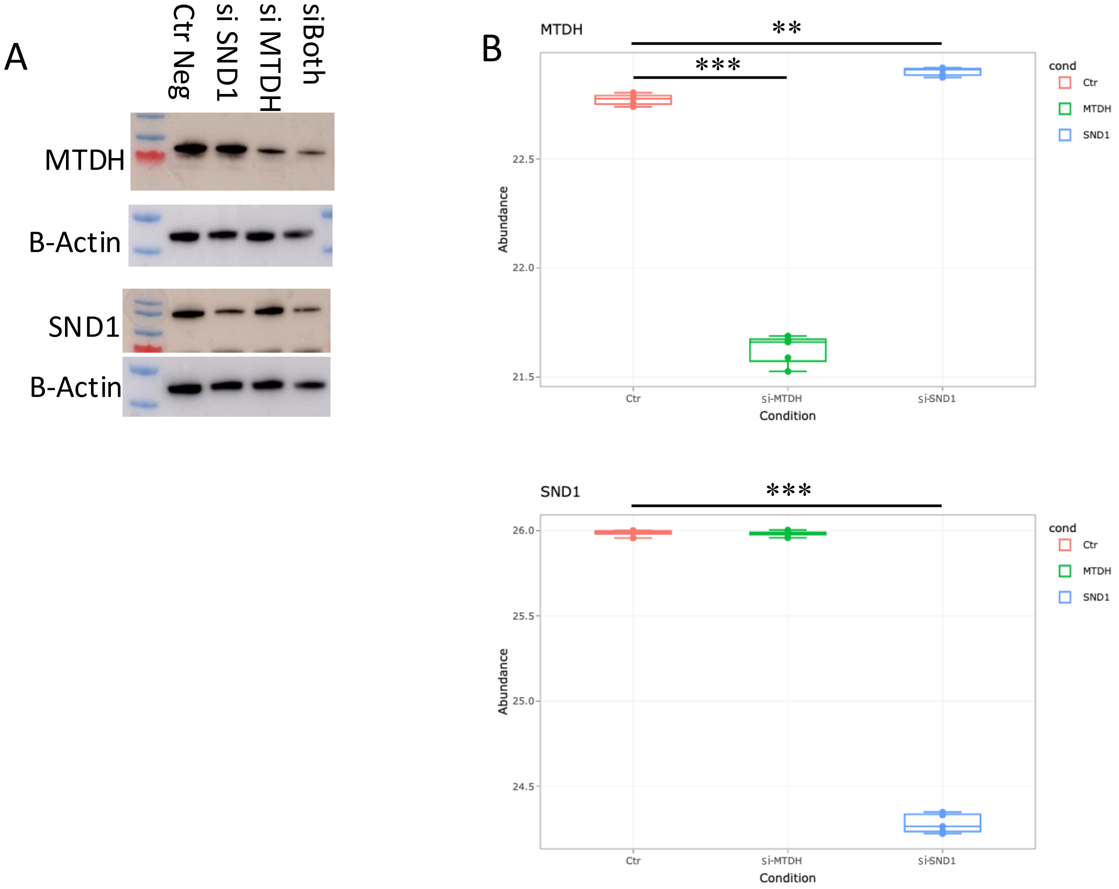


**Supplementary Figure 3.** Transfection validation.
**A)** Western blot analysis using anti-MTDH and anti-SND1 antibodies. The full western blot pictures can be found in Supplementary Figure 10.
**B)** Abundance levels of MTDH and SND1 after silencing, as measured in the proteomics dataset. Abundance values are log2-transformed normalized protein intensity values. Statistical significance of LIMMA test in comparisons with control indicated as follows: $^{**}p < 0.01$, $^{***}p < 0.001$.

# IGROV1

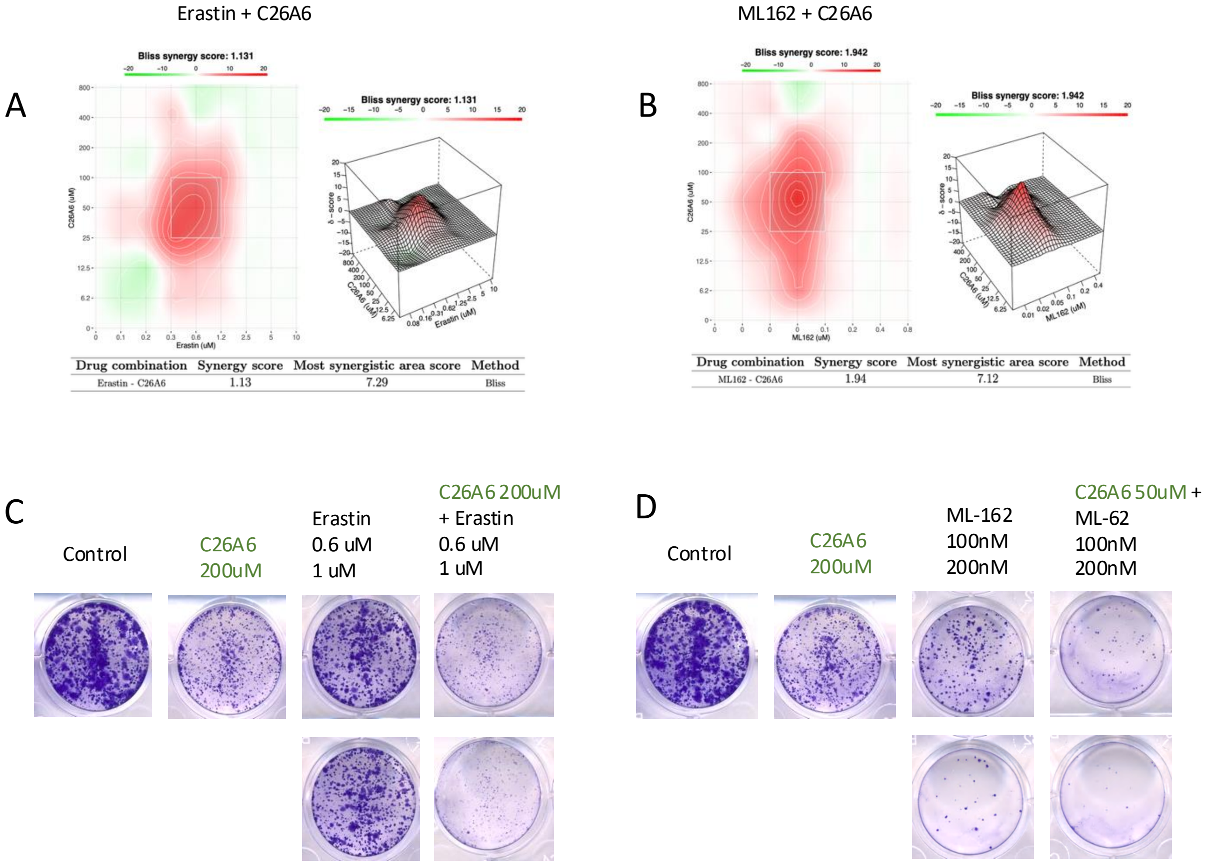


**Supplementary Figure 4.** Synergistic effect on IGROV1 of Erastin or ML-162 with C26A6, and colony formation assay. **A)** Synergy analysis of Erastin combined with C26A6 using cell viability assays over 5 days. **B)** Synergy analysis of ML-162 combined with C26A6 using cell viability assays over 5 days. **C)** Colony formation assay showing the effects of Erastin and C26A6 combination treatment on colony growth after 10 days. **D**) Colony formation assay showing the effects of ML-162 and C26A6 combination treatment on colony growth after 10 days.

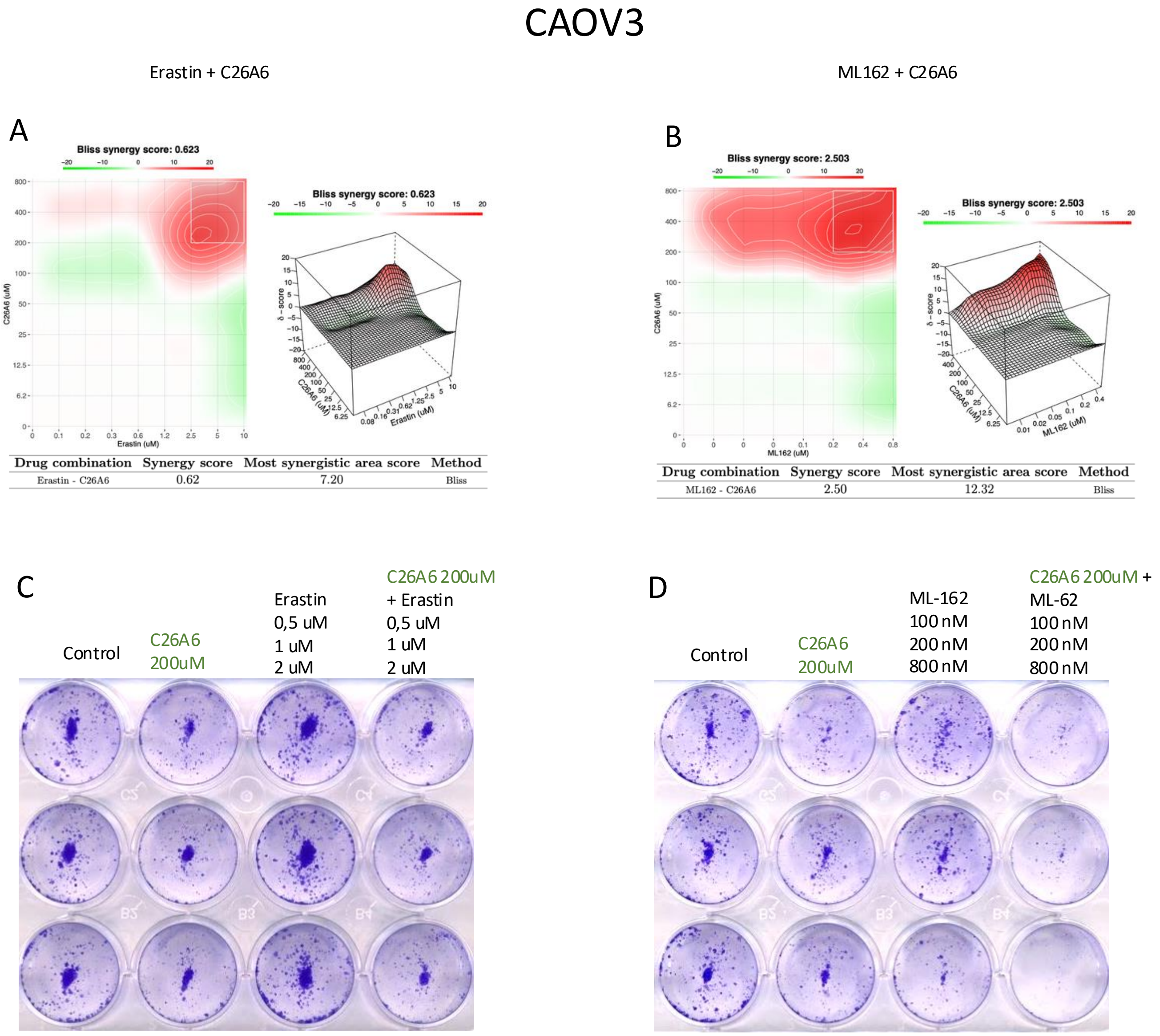


**Supplementary Figure 5.** Synergistic effect on CAOV3 of Erastin or ML-162 with C26A6, and colony formation assay. **A)** Synergy analysis of Erastin combined with C26A6 using cell viability assays over 5 days. **B)** Synergy analysis of ML-162 combined with C26A6 using cell viability assays over 5 days. **C)** Colony formation assay showing the effects of Erastin and C26A6 combination treatment on colony growth after 10 days. **D)** Colony formation assay showing the effects of ML-162 and C26A6 combination treatment on colony growth after 10 days.

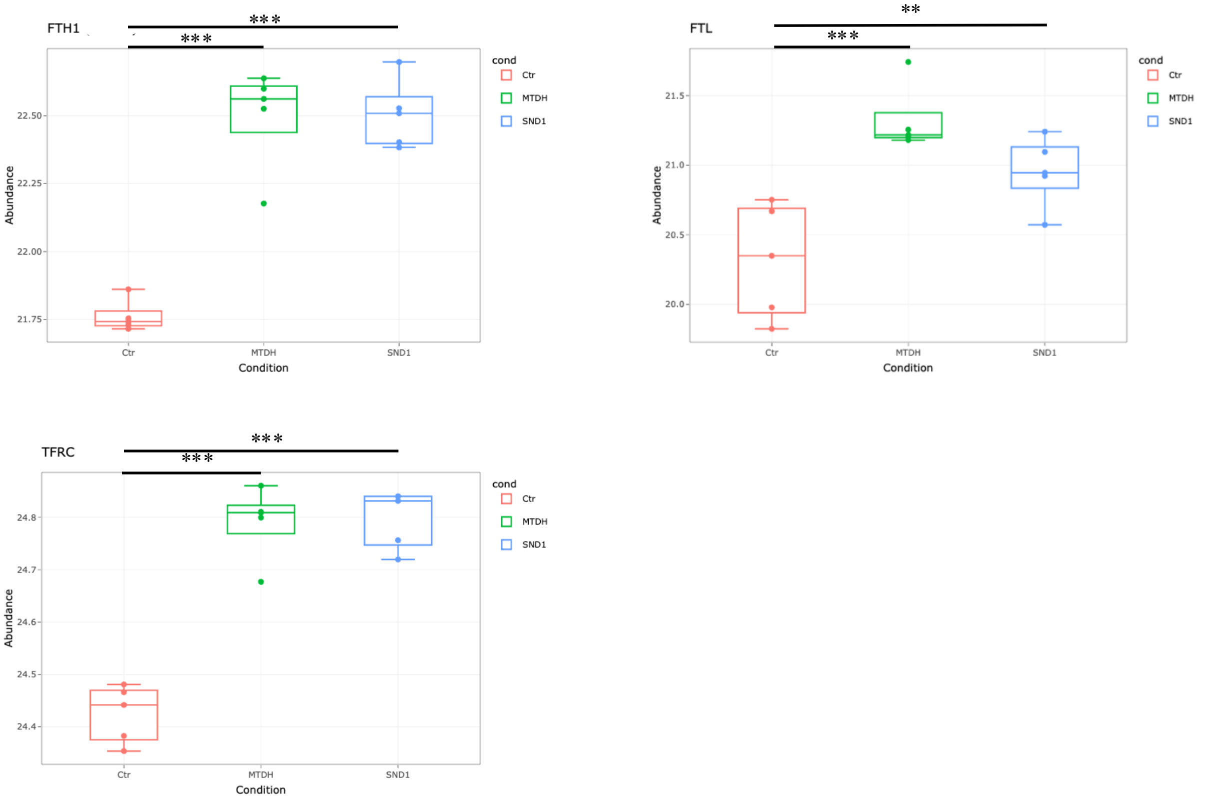


**Supplementary Figure 6.** Expression of FTH1, FTL, and TFRC in MTDH- and SND1-silenced samples compared to control. The Y-axis show the log2-transformed normalized protein abundance values. Statistical significance of LIMMA test in comparisons with controls indicated as follows: ** $p < 0.01$, *** $p < 0.001$.

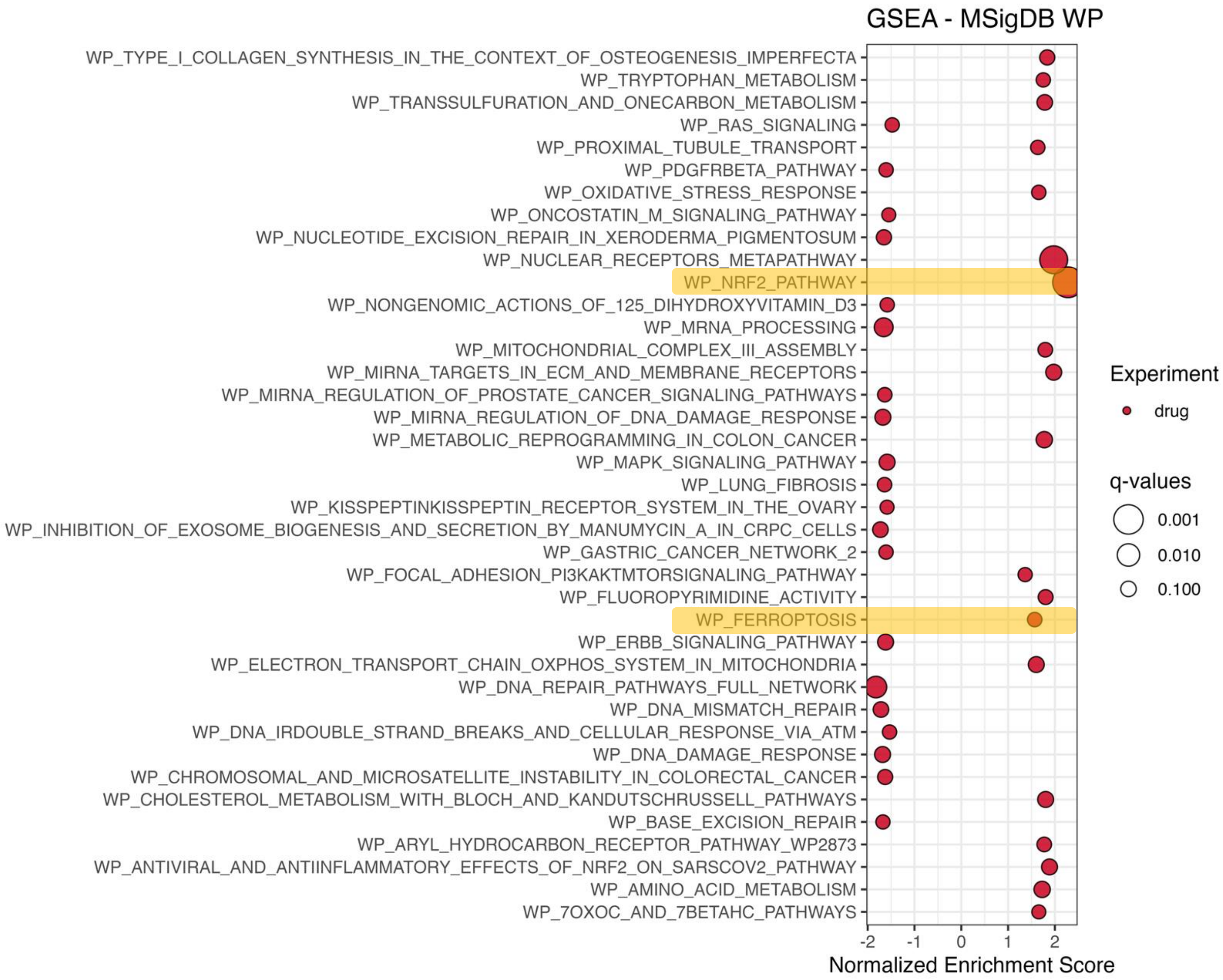


**Supplementary Figure 7.** GSEA using Wikipathways gene sets for treatment with C26A6. NRF2 and ferroptosis gene sets are highlighted.

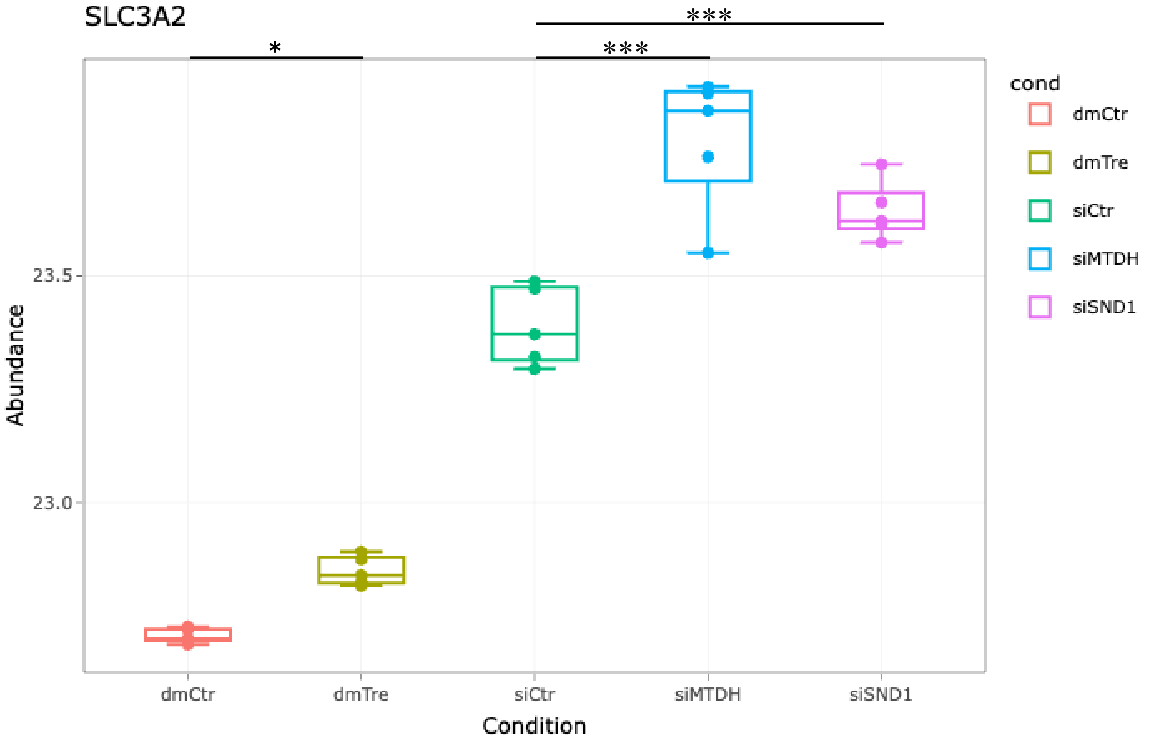


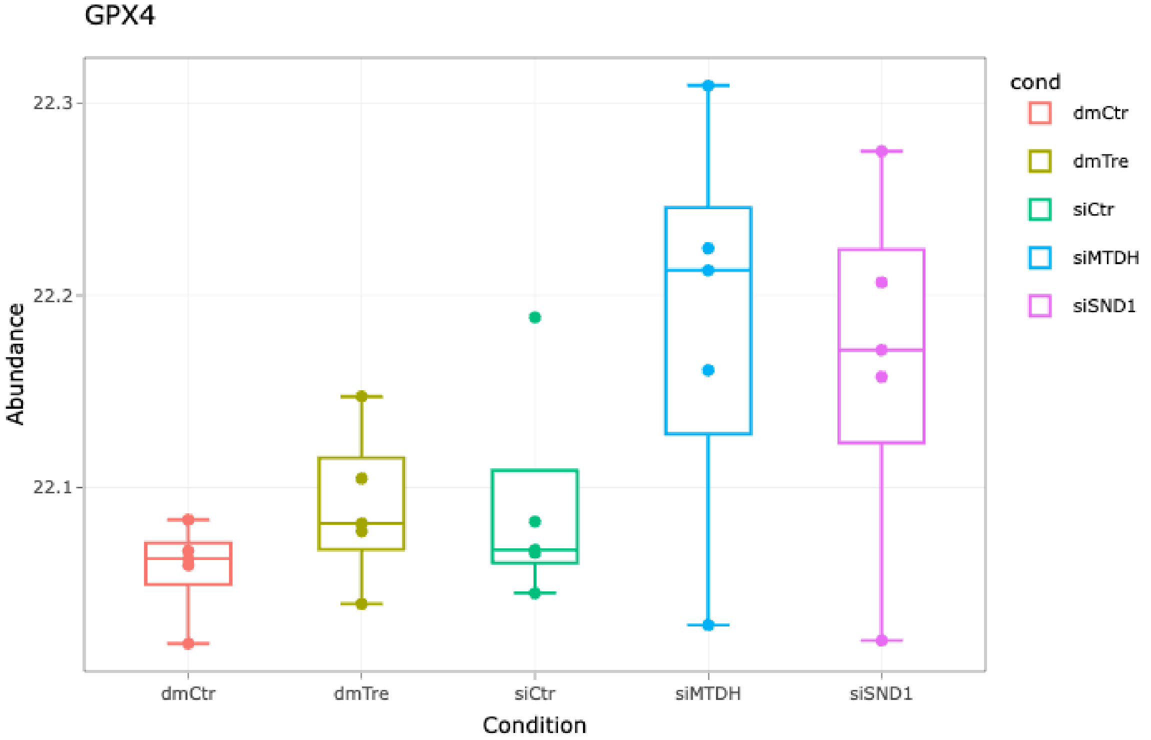


**Supplementary Figure 8.** Expression pattern of GPX4 and SLC3A2 across treatment and transfection samples. The Y-axis shows the log2-transformed normalized abundance value. **dmCtr**: DMSO-treated control. **dmTre**: Treated with 50 µM C26A6. **siCtrl**: Negative control transfection. **siMTDH**: MTDH-silenced transfection. **siSND1**: SND1-silenced transfection. Statistical significance of LIMMA tests in comparison with control indicated as follows: *$p < 0.05$, ***$p < 0.001$. non-significant differences are not indicated.

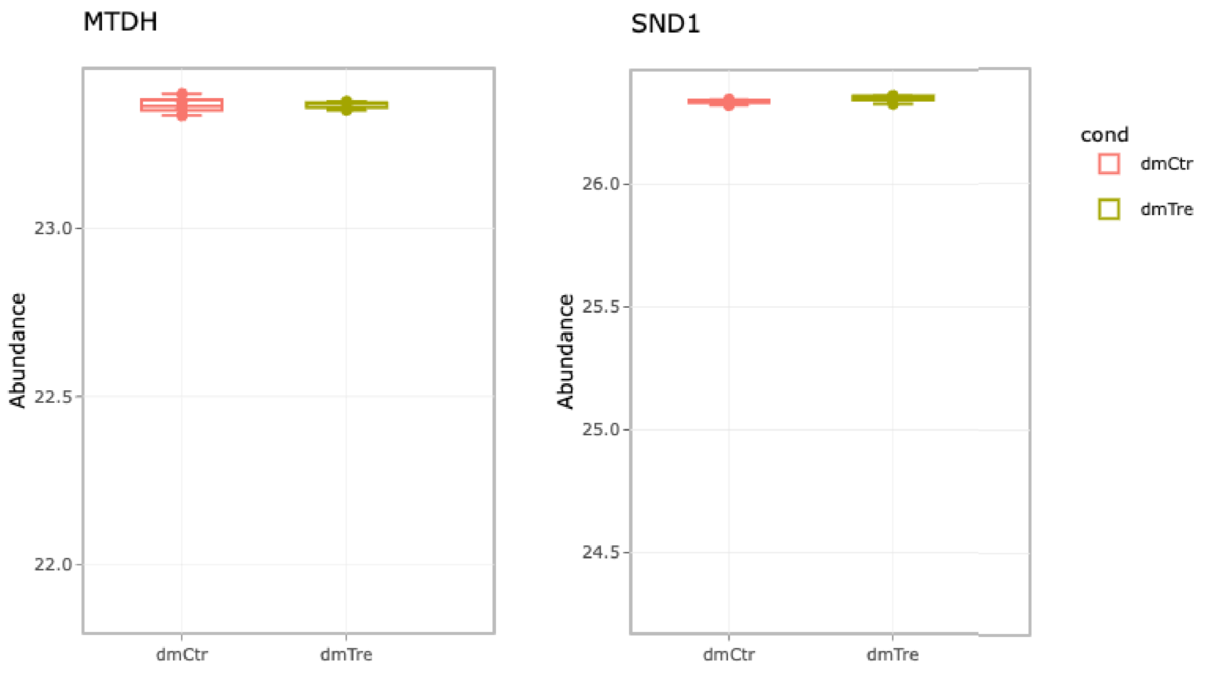


**Supplementary Figure 9.** Expression patterns of MTDH and SND1 after treatment with 50 μM C26A6. dmCtr: DMSO-treated control. dmTre: Treated with 50 μM C26A6. Abundance values are log2-transformed normalized protein abundance values. Statistical significance was assessed using LIMMA, which indicated no significant changes (P<0.05) in SND1 or MTDH after treatment.

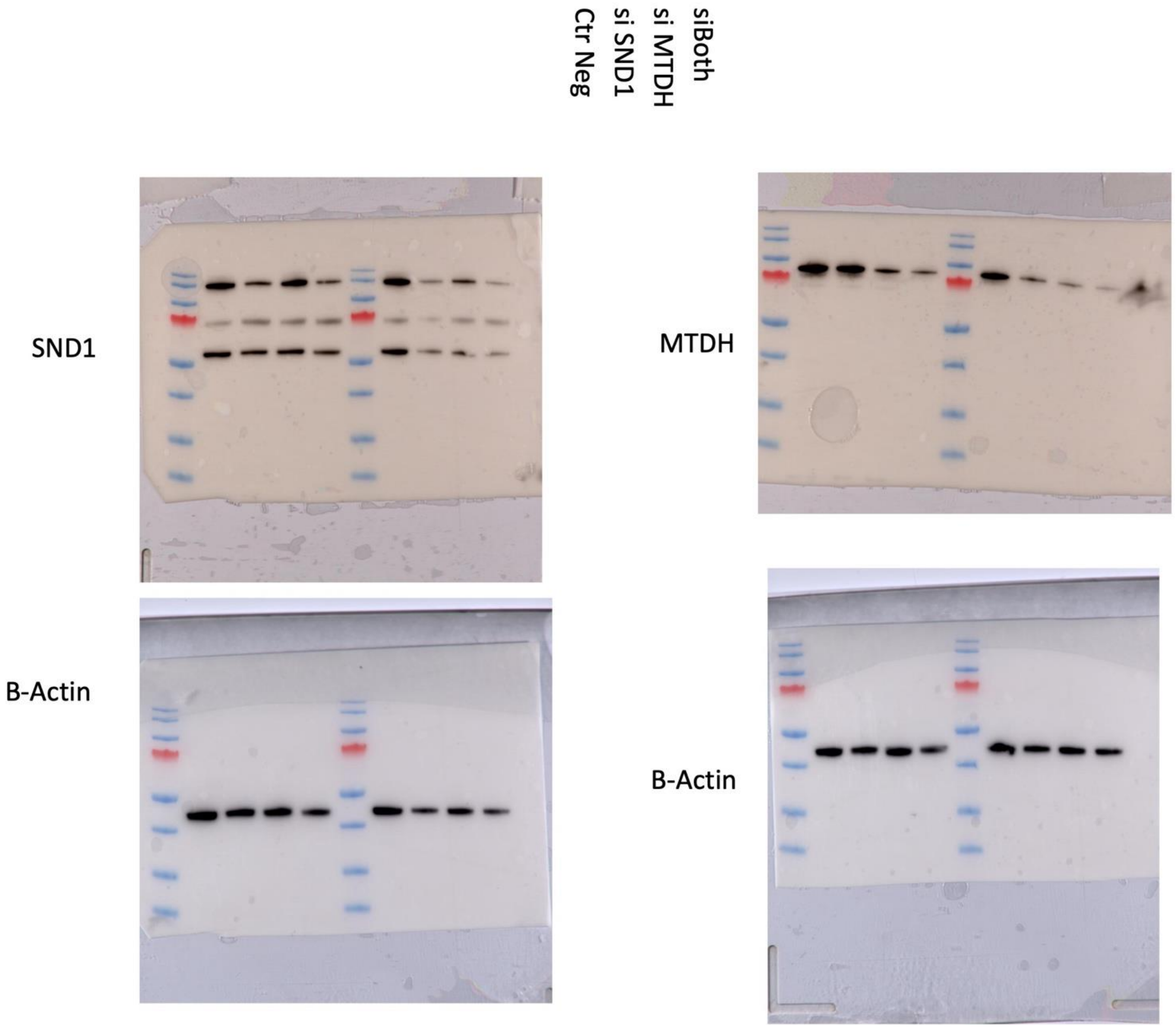


**Supplementary Figure 10.** Full western membrane pictures of western blots in Supplementary Figure 3A. Antibodies are indicated to the left of the pictures, and the loading order is shown above the pictures. The loading was repeated twice, with the replicates between the ladders shown in Supplementary Figure 3.

## Supplementary Table 1. Gene set over-representation analysis following MTDH and SND1 silencing.*

| DB | MTDH | FDRq-value | SND1 | FDRq-value |
|---|---|---|---|---|
| C2: WikiPathways | **VEGFAVEGFR2_SIGNALING** | 7.93 e$^{-16}$ | FRAGILE_X_SYNDROME | 1.03 e$^{-9}$ |
| | **IL18_SIGNALING** | 5.57 e$^{-14}$ | **IL18_SIGNALING** | 2.01 e$^{-9}$ |
| | **FOCAL_ADHESION_PI3KAKTMTORSIGNALING** | 3.73 e$^{-12}$ | COMPLEMENT_AND_COAGULATION_CASCADES | 2.18 e$^{-9}$ |
| | **PI3KAKT_SIGNALING** | 5.02 e$^{-12}$ | **VEGFAVEGFR2_SIGNALING** | 2.18 e$^{-9}$ |
| | METABOLIC_REPROGRAMMING_IN_PANCREATEATIC_CANCER | 1.69 e$^{-10}$ | DENGUE2_INTERACTIONS_WITH_COMPLEMENMENT_AND_COAGULATION_CASCADES | 2.18 e$^{-9}$ |
| | **PLEURAL_MESOTHELIOMA** | 1.96 e$^{-10}$ | **PLEURAL_MESOTHELIOMA** | 7.73 e$^{-8}$ |
| | FOCAL_ADHESION | 1.96 e$^{-10}$ | NETWORK_MAP_OF_SARSCOV2_SIGNALING | 7.73 e$^{-8}$ |
| | PRIMARY_FOCAL_SEGMENTAL_GLOMERULOSCOSCLEROSIS_FSGS | 8.83 e$^{-7}$ | **PI3KAKT_SIGNALING** | 1.61 e$^{-7}$ |
| | FRAGILE_X_SYNDROME | 1.28 e$^{-6}$ | FATTY_ACIDS_AND_LIPOPROTEINS_TRANSPNSPORT_IN_HEPATOCYTES | 8.77 e$^{-7}$ |
| | IRON_METABOLISM_DISORDERS | 1.82 e$^{-6}$ | **FOCAL_ADHESION_PI3KAKTMTORSIGNALING** | 8.77 e$^{-7}$ |
| C5: GO: MF | **PROTEIN_CONTAINING_COMPLEX_BINDINDING** | 8.49 e$^{-18}$ | **OXIDOREDUCTASE_ACTIVITY** | 1.08 e$^{-16}$ |
| | **CELL_ADHESION_MOLECULE_BINDING** | 2.77 e$^{-17}$ | **PROTEIN_CONTAINING_COMPLEX_BINDINDING** | 1.36 e$^{-14}$ |
| | **PURINE_NUCLEOTIDE_BINDING** | 4.83 e$^{-15}$ | **ENZYME_REGULATOR_ACTIVITY** | 1.36 e$^{-14}$ |
| | **MOLECULAR_ADAPTOR_ACTIVITY** | 6.06 e$^{-15}$ | **CELL_ADHESION_MOLECULE_BINDING** | 6.79 e$^{-12}$ |
| | **ENZYME_REGULATOR_ACTIVITY** | 8.23 e$^{-15}$ | **SIGNALING_RECEPTOR_BINDING** | 1.04 e$^{-10}$ |
| | **ADENYL_NUCLEOTIDE_BINDING** | 9.43 e$^{-14}$ | **PURINE_NUCLEOTIDE_BINDING** | 5 e$^{-10}$ |
| | **OXIDOREDUCTASE_ACTIVITY** | 1.14 e$^{-11}$ | **MOLECULAR_ADAPTOR_ACTIVITY** | 8.58 e$^{-10}$ |
| | **SIGNALING_RECEPTOR_BINDING** | 2.5 e$^{-10}$ | **ADENYL_NUCLEOTIDE_BINDING** | 4.36 e$^{-9}$ |
| | KINASE_BINDING [794] | 4.69 e$^{-10}$ | TRANSITION_METAL_ION_BINDING | 3.28 e$^{-8}$ |
| | CADHERIN_BINDING [335] | 1.36 e$^{-9}$ | PEPTIDASE_REGULATOR_ACTIVITY | 3.46 e$^{-8}$ |

*Gene set overlap analysis using the top 500 up-regulated proteins after filtering at FDR < 0.01.
DB = Database

**The bold text indicates pathways that are present in both MTDH and SND1 silenced samples.**

**Supplementary Table 2. Phosphoenrichment analysis of kinases in MTDH- or SND1-silenced samples.**
**A)** Downregulated kinases. **B)** Upregulated kinases. Kinases highlighted in color indicate those shared between MTDH and SND1 silenced samples. Left MTDH, right SND1

A

| KINASE-iKiP | Signature.set.size | Signature.set.overlap.percent.MTDH.ctrl | fdr.pvalue.MTDH.ctrl | NES.MTDH-ctrl |
|---|---|---|---|---|
| CSNK2A2.CK2A2 | 323 | 15,8 | 0,00314615 | -7,6074 |
| CSNK2A1.CK2A1 | 100 | 23 | 0,00314615 | -6,7358 |
| PIK3R5 | 32 | 25 | 0,00314615 | -4,9565 |
| ACVR1.ALK2 | 157 | 9,6 | 0,00314615 | -4,7931 |
| GRK7 | 109 | 10,1 | 0,00314615 | -4,3572 |
| BMPR1B | 91 | 11 | 0,01800538 | -4,027 |
| TGFBR1 | 73 | 12,3 | 0,01704688 | -3,8896 |
| TLK2 | 125 | 14,4 | 0,02615102 | -3,7334 |
| CHUK.IKKA | 82 | 13,4 | 0,02615102 | -3,5929 |
| CSNK1D.CK1D | 206 | 10,7 | 0,04171767 | -3,4577 |
| NEK7 | 197 | 6,6 | 0,04295735 | -3,3539 |
| CSNK1G2 | 155 | 9 | 0,04983691 | -3,2189 |
| PLK1 | 156 | 9 | 0,04730101 | -3,2183 |

| KINASE-iKiP | Signature.set.size | Signature.set.overlap.percent.SND1.ctrl | fdr.pvalue.SND1.ctrl | NES.SND1-ctrl |
|---|---|---|---|---|
| CSNK2A2.CK2A2 | 323 | 15,8 | 0,00141034 | -10,3673 |
| CSNK2A1.CK2A1 | 100 | 23 | 0,00141034 | -7,8343 |
| MAPK10.JNK3 | 164 | 36 | 0,00141034 | -6,5632 |
| MAPK12 | 430 | 37,2 | 0,00141034 | -6,2402 |
| MAPK13 | 413 | 38 | 0,00141034 | -6,0163 |
| MAPK9.JNK2 | 258 | 33,7 | 0,00141034 | -5,9971 |
| MAPK1.ERK2 | 448 | 37,7 | 0,00141034 | -5,4998 |
| PIK3R5 | 32 | 25 | 0,00141034 | -5,4467 |
| ACVR1.ALK2 | 157 | 9,6 | 0,00141034 | -5,4223 |
| GRK7 | 109 | 10,1 | 0,00141034 | -5,258 |
| GRK5 | 169 | 8,3 | 0,00141034 | -5,1161 |
| CDK1-CCNB1 | 253 | 44,3 | 0,00141034 | -4,9647 |
| MAPK3.ERK1 | 304 | 38,2 | 0,0038736 | -4,9216 |
| PLK3 | 233 | 8,2 | 0,00223812 | -4,5932 |
| TGFBR1 | 73 | 12,3 | 0,00141034 | -4,4023 |
| CDK5-CDK5R1p35 | 186 | 44,1 | 0,00223812 | -4,2625 |
| MAPK8.JNK1 | 380 | 36,3 | 0,0038736 | -4,2451 |
| BMPR1B | 91 | 11 | 0,0038736 | -4,1846 |
| CSNK1D.CK1D | 206 | 10,7 | 0,00223812 | -4,1774 |
| CDK2-CCNE1 | 140 | 42,1 | 0,00535944 | -4,159 |
| CSNK1E.CK1E | 114 | 10,5 | 0,00223812 | -4,0336 |
| MAPK14 | 292 | 30,8 | 0,01764218 | -3,9091 |
| LATS2 | 94 | 20,2 | 0,01433255 | -3,8725 |
| CSNK1G2 | 155 | 9 | 0,01668066 | -3,7171 |
| CDK2-CCNA2 | 151 | 46,4 | 0,03513194 | -3,5444 |
| CDK4-CCND1 | 32 | 37,5 | 0,02920413 | -3,4905 |
| TLK2 | 125 | 14,4 | 0,04363539 | -3,344 |
| TLK1 | 183 | 11,5 | 0,03795141 | -3,3191 |
| MAPK7.ERK5 | 115 | 22,6 | 0,04148435 | -3,3008 |
| MAPK11 | 212 | 30,2 | 0,04947238 | -3,1832 |

B

| KINASE-iKiP | Signature.set.size | Signature.set.overlap.percent.MTDH.ctrl | fdr.pvalue.MTDH.ctrl | NES.MTDH-ctrl |
|---|---|---|---|---|
| PRKCB | 159 | 23,9 | 0,00314615 | 5,8517 |
| MAP2K4 | 41 | 51,2 | 0,00314615 | 5,4219 |
| CGK2 | 83 | 24,1 | 0,00314615 | 5,4064 |
| MAP2K7 | 28 | 39,3 | 0,00314615 | 4,7053 |
| MAPKAPK2 | 159 | 37,1 | 0,01266823 | 4,4693 |
| PRKD3 | 112 | 23,2 | 0,01266823 | 4,2689 |
| SGK3 | 143 | 25,2 | 0,00873633 | 4,2162 |
| CDK9-CCNK | 115 | 48,7 | 0,01172323 | 3,9746 |
| SRPK2 | 24 | 62,5 | 0,01172323 | 3,9572 |
| SRPK3 | 24 | 58,3 | 0,00873633 | 3,9437 |
| PRKD2 | 61 | 41 | 0,02565201 | 3,9208 |
| MAP3K2 | 40 | 35 | 0,01704688 | 3,8906 |
| MAPKAPK5 | 141 | 31,9 | 0,04026664 | 3,5964 |
| MAPK15.ERK7 | 65 | 33,8 | 0,0377864 | 3,5534 |
| CHEK1 | 107 | 12,1 | 0,0377864 | 3,5389 |
| ERBB2 | 84 | 41,7 | 0,04630062 | 3,5111 |
| CHEK2 | 34 | 20,6 | 0,01925476 | 3,4474 |
| SIK1 | 184 | 22,8 | 0,04630062 | 3,4047 |
| NLK | 131 | 30,5 | 0,04983691 | 3,3955 |
| PRKD1 | 66 | 28,8 | 0,04730101 | 3,2953 |

| KINASE-iKiP | Signature.set.size | Signature.set.overlap.percent.SND1.ctrl | fdr.pvalue.SND1.ctrl | NES.SND1-ctrl |
|---|---|---|---|---|
| PRKACA | 190 | 22,6 | 0,00141034 | 5,5747 |
| SRPK3 | 24 | 58,3 | 0,00141034 | 4,7644 |
| SRPK2 | 24 | 62,5 | 0,00141034 | 4,7406 |
| SIK2 | 195 | 22,6 | 0,00141034 | 4,6951 |
| PRKD2 | 61 | 41 | 0,0038736 | 4,5292 |
| HIPK1 | 47 | 59,6 | 0,00223812 | 4,4895 |
| SGK1 | 82 | 34,1 | 0,00223812 | 4,47 |
| AKT1 | 71 | 29,6 | 0,00141034 | 4,4569 |
| MARK2 | 167 | 24 | 0,00535944 | 4,3686 |
| CGK2 | 83 | 24,1 | 0,0038736 | 4,3484 |
| PRKCB | 159 | 23,9 | 0,00981796 | 4,0336 |
| AKT2 | 70 | 21,4 | 0,01651524 | 3,6482 |
| PRKX | 67 | 26,9 | 0,01651524 | 3,6476 |
| CDK9-CCNK | 115 | 48,7 | 0,03768308 | 3,5234 |
| SGK3 | 143 | 25,2 | 0,04072083 | 3,3509 |
| TSSK1 | 233 | 15,5 | 0,04313556 | 3,2529 |
| PRKD3 | 112 | 23,2 | 0,04947238 | 3,1916 |
| TSSK2 | 73 | 12,3 | 0,04930681 | 3,0468 |
